# Increased Soil Nitrogen Availability Suppresses Annual Soil Respiration in Mixed Temperate Forests Regardless of Acidification

**DOI:** 10.1111/gcb.70140

**Published:** 2025-04-02

**Authors:** David W. Frey, Eden Kebede, Jed P. Sparks, Timothy J. Fahey, Christine L. Goodale

**Affiliations:** ^1^ Department of Ecology and Evolutionary Biology Cornell University Ithaca New York USA; ^2^ Department of Natural Resources and the Environment Cornell University Ithaca New York USA

**Keywords:** belowground carbon allocation, belowground carbon partitioning, heterotrophic respiration, nitrogen addition, soil acidification, soil respiration

## Abstract

Soil respiration (Rsoil) is the second largest terrestrial carbon (C) flux, and therefore, it is imperative to understand and quantify its responses to global environmental change. Rsoil consists of two component CO_2_ fluxes: autotrophic respiration from the metabolic activity of roots (Ra_‐root_) and heterotrophic respiration (Rh) derived from the metabolic activity of mycorrhizal fungi and microbial decomposition of detritus, soil organic matter, and rhizodeposits. Increased nitrogen (N) availability often reduces Rsoil in forest ecosystems, but it remains unclear which contributing fluxes govern Rsoil responses and if suppression of Rsoil results from increased N availability itself or from the tendency of added N to acidify soil. Here, we address these uncertainties in a long‐term, large‐scale factorial N × pH experiment in six temperate forest stands in central New York, USA. We anticipated that increasing soil N availability would decrease plant belowground C allocation and related root‐associated respiration and that soil acidification would suppress microbial decomposition, thereby reducing Rh. We found that both acidifying and deacidifying N additions suppressed annual Rsoil by 19% and 13%, respectively (−1.8 Mg C ha^−1^ year^−1^ overall), but acidification (from pH 4.67 to 4.22) alone did not detectably affect this flux. Annual Rsoil decreased steeply (*R* ^2^ = 0.66, *p* < 0.001) as soil N availability increased. Nitrogen additions generally suppressed Rh, especially in the forest floor (−34%), whereas the effects of acidification alone varied by soil depth, with substantial suppression in the forest floor (−33%) partially offset by stimulation at depth. A novel partitioning of Rsoil component responses suggests that N additions suppressed root‐associated respiration by ~1.1 Mg C ha^−1^ year^−1^ (62% of the Rsoil suppression), while acidification alone had no effect. Our findings demonstrate that soil N availability, not soil pH, is the predominant biogeochemical control over Rsoil in these temperate forests, with larger responses of plant‐driven C fluxes than microbial‐driven C fluxes.

## Graphical abstract

We investigated how soil nitrogen availability and acidity independently or interactively affect soil respiration (Rsoil) and its plant‐associated (R_‐root‐assoc_) and decomposer‐driven (Rh) component fluxes in mixed temperate forests in central New York, USA. Experimentally increased soil nitrogen availability reduced Rsoil (−1.8 Mg C ha^−1^ year^−1^) by suppressing both R_‐root‐assoc_ (~1.1 Mg C ha^−1^ year^−1^) and Rh‐driven (~0.7 Mg C ha^−1^ year^−1^) contributions to Rsoil, while acidification had no effect on Rsoil but suppressed Rh in surface soils. Our findings demonstrate that soil N availability, not soil pH, is the predominant biogeochemical control over Rsoil in these temperate forests.

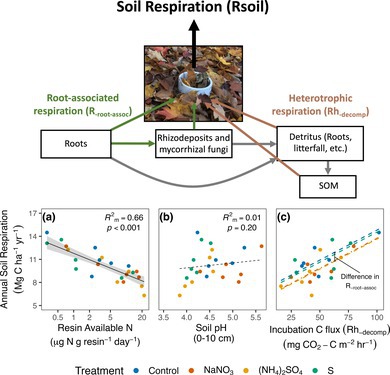

## 1 Introduction

Soil respiration (Rsoil) plays an enormous role in the global carbon (C) cycle: only one other terrestrial C flux—gross primary production—is larger, and Rsoil exceeds fossil fuel emissions by nearly tenfold (Friedlingstein et al. 2022; Lei et al. 2021). Consequently, factors that alter Rsoil rates can exert strong effects on the global C cycle and climate. Temperature is the predominant factor governing respiration rates at the global scale (Hursh et al. 2017; Raich et al. 2002), but Rsoil can be sensitive to a variety of other factors, such as nutrient availability (Bowden et al. 2004; Knorr et al. 2024; Janssens et al. 2010; Kang et al. 2016) and pH (Chen et al. 2014; Lu et al. 2022; Meng et al. 2019). Given that natural ecological processes, underlying geology, industrial activities, and agricultural practices can drive wide variability in these soil properties across terrestrial systems, accurately predicting Rsoil depends on understanding how pH and nutrient availability independently or interactively drive this globally important flux.

Nitrogen (N) limitation is common in terrestrial ecosystems (LeBauer and Treseder 2008; Vitousek and Howarth 1991), and meta‐analyses show that increased N availability often (Janssens et al. 2010; Zhou et al. 2014), but not always (Liu and Greaver 2010; Liu et al. 2023), suppresses Rsoil in forests. However, there is considerable uncertainty about the roles of changes in nutrient availability versus soil free acidity in Rsoil responses (Averill and Waring 2018; Janssens et al. 2010) because soil acidification often co‐occurs with increased N availability (Tian and Niu 2015; van Breemen et al. 1983). Acid deposition is a well‐known example of this phenomenon, where atmospheric N inputs can acidify ecosystems, an effect intensified by concurrent sulfur (S) deposition (van Breemen et al. 1984; Driscoll et al. 2001). Experimental N additions and N fertilization also often decrease soil pH, as many apply ammonium‐based fertilizers (Liu and Greaver 2010; Guo et al. 2010; Yue et al. 2016) that contribute to acidification by both plant‐ and microbially mediated processes (van Breemen et al. 1983). Therefore, common approaches that examine how increased N availability affects Rsoil may confound nutrient and pH effects.

Rsoil consists of several component fluxes (Equation 1) that are difficult to distinguish operationally: (1) root respiration (Ra_‐root_) that results from the metabolic activity of plant tissues and can only be estimated by isolating roots from soil (with consequent artifacts); (2) heterotrophic respiration (Rh_‐decomp_) driven by the breakdown of dead organic matter by microbial decomposers, which can be estimated in laboratory incubation assays of root‐free soil samples (Billings and Ziegler 2008; Cleveland and Townsend 2006; Kutsch et al. 2010); (3) respiration by fungal endophytes, especially mycorrhizal fungi, both intra‐ and extra‐matrical (Rh_‐myco_); and (4) microbial decomposition of root exudates and other rhizodeposits (Rh_‐rhizo_).

(1) $$\text{Rsoil}={\mathrm{Ra}}_{-\text{root}}+{\mathrm{Rh}}_{-\text{decomp}}+{\mathrm{Rh}}_{-\text{myco}}+{\mathrm{Rh}}_{-\text{rhizo}}$$

The latter two processes are clearly major components (Han et al. 2021; Hawkins et al. 2023; Kuzyakov and Larionova 2006) of total soil respiration that are challenging to quantify (Heinemeyer et al. 2007; Kuzyakov and Larionova 2006). For the present study, we evaluated three “operational” components of soil respiration and their responses to changes in N availability and soil pH. We directly measured two fluxes, Rsoil, the flux of CO_2_ from the soil surface, and Rh_‐decomp_, defined here as CO_2_ emission in aerobic laboratory assays of sieved soil samples (note that Rh_‐decomp_ may include some Rh_‐rhizo_). We then use a novel partitioning approach to estimate how N availability and soil pH affect the combined flux of the three soil respiration components not directly measured here (Ra_‐root_, Rh_‐myco_, and Rh_‐rhizo_). We term this combined flux “root‐associated respiration” (R_‐root‐assoc_, Equation 2). This operational approach is necessitated by the challenge of directly distinguishing these components in situ.

(2) $$R_{-\text{root}-\text{assoc}}={\mathrm{Ra}}_{-\text{root}}+{\mathrm{Rh}}_{-\text{myco}}+{\mathrm{Rh}}_{-\text{rhizo}}$$

Changes in each of these operationally defined Rsoil component fluxes (Rh_‐decomp_, R_‐root‐assoc_) have different implications for ecosystem C storage and productivity, as shifts in plant belowground C allocation that affect R_‐root‐assoc_ should alter the amount of photosynthate available for aboveground biomass production, whereas changes in Rh_‐decomp_ can impact soil C storage. Hence, an integrated understanding of how Rsoil and its component fluxes respond to environmental changes can better inform how these perturbations will impact ecosystem C balance.

Several autotrophic and heterotrophic mechanisms may contribute to observed responses of Rsoil to increased N availability in forests, and although suppression is common (Janssens et al. 2010; Zhou et al. 2014), various countervailing mechanisms may offset to yield neutral responses (Liu and Greaver 2010; Liu et al. 2023). For example, increased N availability may stimulate specific root respiration because N‐rich roots typically exhibit higher respiration rates than nitrogen‐poor roots (Burton et al. 2002; Zogg et al. 1996); however, N availability can also affect fine root biomass and production (Li et al. 2015; Peng et al. 2017) with consequent effects on the overall Ra_‐root_ flux. Additionally, soil N enrichment should reduce Rh_‐myco_ and Rh_‐rhizo_ because increased N availability decreases the need for plants to allocate C to mycorrhizae (Carrara et al. 2022; Treseder 2004) and to fuel rhizosphere saprotroph activity (Phillips and Fahey 2007) to access nutrients. These effects may explain why increased N availability can suppress total belowground C allocation of plants (Bae et al. 2015; Eastman et al. 2021). Further, increased N availability can suppress microbial oxidative exoenzyme activity (Bonner et al. 2019; Chen et al. 2018), which can reduce Rh_‐decomp_ by slowing lignin decay (Frey et al. 2014; Hasegawa et al. 2021). Together, these mechanisms may contribute to often substantial reductions in forest Rsoil with increasing soil N availability.

Soil acidification can also suppress Rsoil. Microbial biomass and activity are often lower in more acidic soils (Anderson and Domsch 1993; Bååth and Anderson 2003; Chen, Li, et al. 2016; Francis 1982), an observation that underpins the proposed Carbon, Acidity, and Mineral Protection (CAMP) framework for soil organic matter (Averill and Waring 2018). This framework posits that increased soil N should stimulate Rh_‐decomp_ when decoupled from acidification and only reduce Rh_‐decomp_ when combined with strong soil acidification, owing to the impacts of acidification on soil microbial activity. Two recent meta‐analyses (Lu et al. 2022; Meng et al. 2019) provide some support for this hypothesis, although they span very large pH ranges (~3.0–8.0).

We are aware of only two experiments in forests that have independently manipulated soil pH and N availability, and both used relatively small plot sizes (9–100 m^2^) that are unlikely to capture plant‐driven responses. One study suggests that acidification predominantly drives suppressed Rsoil under N enrichment (Li et al. 2018), while the other suggests that acidification alone, and not increased N availability, reduces the flux (Oulehle et al. 2018). Small plot sizes have the advantage of easier replication, and they may serve well to capture many soil microbial responses to experimental manipulations, but tree root systems can extend over 20 m beyond their stems (Lyford and Wilson 1964; Meinen et al. 2009). Hence, in small forested plots, only a fraction of trees' root systems directly experience experimental treatment effects, which limits the likelihood of inducing responses to plant‐driven belowground C fluxes. Moreover, roots and mycorrhizal hyphae can proliferate in small, nutrient‐rich patches (Chen, Koide, et al. 2016; Hodge 2004, 2006), a process that could introduce artifacts to small plots with soil conditions that may attract (increased N availability) or repel (acidification) local root and mycorrhizal activity. Given that root‐associated respiration can constitute a considerable fraction of Rsoil (Abramoff and Finzi 2016; Kelting et al. 1998), it is imperative to capture these plant‐driven processes to understand how Rsoil responds to changes in N availability and soil acidity.

Here, we seek to understand the roles of soil pH and N availability in driving Rsoil in temperate forests in central New York, USA, using a replicated long‐term, large‐plot N × pH manipulation study. We anticipated that increasing soil N availability, regardless of pH change, would reduce belowground C allocation by plants, whereas acidification without N might increase carbon allocated belowground to acquire nutrients and thereby stimulate higher Rsoil. We expected that a de‐acidifying N addition would stimulate decomposition (Rh_‐decomp_) by N‐limited microbes, especially in the C‐rich forest floor, as proposed by Averill and Waring (2018). In contrast, we expected that acidifying soils would suppress Rh_‐decomp_. Collectively, the effects of N addition and pH on Rsoil should reflect the net impact of their influence on these plant and microbial processes.

## 2 Materials and Methods

### 2.1 Site Description and Experimental Design

The study was conducted in mixed temperate forests in central New York, USA, at three different sites within 20 km of Ithaca, NY (Bald Hill, 42°21.501′, −76°22.536′; Carter Creek, 42°20.116′, −76°40.038′; and Mount Pleasant, 42°28.036′, −76°23.015′). Each site contains two co‐located stand types: primary forests that were never cleared for agriculture and had only limited harvest history (Flinn and Marks 2007; Marks 1995), and secondary, post‐agricultural forests, which were approximately 90 years old at the beginning of the experiment. Tree species composition varied among stands. Primary forests were dominated by mid‐ and late‐successional hardwoods, including sugar maple ( *Acer saccharum* ), white ash ( *Fraxinus americana* ), American beech ( *Fagus grandifolia* ), red oak ( *Quercus rubra* ), and red maple ( *A. rubrum* ). In contrast, in the secondary stands, early‐ and mid‐successional species, including red maple, red oak, white ash, white pine *(Pinus strobus)*, and bigtooth aspen ( *Populus grandidentata* ) were dominant. Tree species with arbuscular mycorrhizal associations (ash and both maples) made up 18%–98% of total stand biomass across our study plots; tree species that typically form ectomycorrhizal associations (beech, oak, pine, and aspen) made up the other 2%–82%. All sites fall within the Köppen‐Geiger warm‐summer humid continental climate zone (Beck et al. 2018). The mean annual temperature at a weather station near Ithaca, NY (1991–2020) is 7.9°C, and mean annual precipitation is 973 mm (Game Farm Rd. Weather Station, NRCC 2024). Soils are composed of acidic channery silt loam Inceptisols developed on glacial till and underlain by Devonian shale (Soil Survey Staff, 2024). Background atmospheric N and S deposition (total wet and dry forms, including NH_3_) at a nearby Environmental Protection Agency Clean Air Status and Trends Network (CASTNET) site have declined substantially over the past 20 years, from 13.4 kg N ha^−1^ year^−1^ and 13.0 kg S ha^−1^ year^−1^ in 2000 to 8.9 kg N ha^−1^ year^−1^ and 1.7 kg S ha^−1^ year^−1^ in 2021 (CASTNET 2024).

Experimental fertilizer additions were initiated in 2011 in three plots within each stand and paired with an unfertilized control (4 plots/stand × 2 stands/site × 3 sites = 24 plots). Plots are 40 m × 40 m for plant and soil measurements, with an additional 10 m perimeter buffer treated to ensure fertilization of the majority of root systems of in‐plot trees. Treatments consist of sodium nitrate (NaNO_3_), ammonium sulfate ((NH_4_)_2_SO_4_), and elemental sulfur (S) additions, with N application rates of 50 kg N ha^−1^ year^−1^ for both N treatments and S application rates of 54 kg S ha^−1^ year^−1^ for both S treatments. Treatments were selected for their abilities to increase (NaNO_3_) or decrease ((NH_4_)_2_SO_4_, S) soil pH, and they correspond with high levels of deposition observed in parts of Europe in the late 20th century (Dise and Wright 1995; Kolář et al. 2015) and modern deposition rates in eastern China (Zhou et al. 2023). For the first 7 years of the experiment (2011–2017), fertilizer applications were distributed across three separate dates in spring and summer, whereas resource constraints required shifting applications to occur as a single dose since 2018. In 2019, soil erosion in the control plot in the primary forest stand at Mount Pleasant necessitated its replacement with a new plot that directly abuts the former control.

### 2.2 Soil Respiration Measurements

In June 2020, five Rsoil collars (10.2 cm diameter PVC pipes cut to 10 cm length) were inserted 3–4 cm in all 24 plots, placing one collar in a fixed position within 5 m of each of the four corners and one near the plot's center. We measured Rsoil using a LI‐6400 Portable Photosynthesis System equipped with a 6400‐09 soil chamber (LI‐COR Bioscience, Lincoln, Nebraska, USA). We measured Rsoil 5–6 times per plot during the snow‐free season between October 2020 and October 2021. Most plots were measured in October 2020, and April, June, July, August, and October 2021, but an equipment malfunction prevented measurements at one site (8 plots) in July 2021. On each measurement date, CO_2_ flux was measured four times per collar, and all measurements per collar on each date were averaged to yield one mean flux per collar per date. We measured soil temperature at 10 cm depth concurrent with flux measurements using the LI‐6400 temperature probe, and soil volumetric water content to 10 cm using a Campbell Scientific HydroSense II TDR probe (Campbell Scientific, Logan, Utah, USA).

We scaled field measurements of Rsoil to annual fluxes using the relationship between soil temperature and measured Rsoil, fitted as a first‐order exponential equation (Eastman et al. 2021; Oulehle et al. 2018; Perez‐Quezada et al. 2016; van't Hoff 1898):

(3) $$\text{Rsoil}=a*e^{\mathrm{bt}},$$

where Rsoil is the soil respiration flux (μmol CO_2_ m^−2^ s^−1^), t is soil temperature at 10 cm depth (°C), and a and b are fitted parameters. We estimated annual Rsoil from November 1, 2020, to October 31, 2021, using plot‐level relationships between Rsoil and temperature from Equation (3) and continuous hourly measurements of soil temperature (Figure S1) at each of the three sites (see Supporting Information Methods for details).

### 2.3 Heterotrophic Respiration (Rh_‐decomp_) Measurements From Soil Incubations

Measurements of CO_2_ released during soil incubations in the lab are a standard method for operationally assessing soil heterotrophic respiration (Billings and Ziegler 2008; Cleveland and Townsend 2006; Kutsch et al. 2010). We measured CO_2_ fluxes from 24‐h laboratory incubations of soils collected over June–July 2022 from three soil depths (forest floor, 0–3 cm, 3–10 cm; 5 samples per soil layer per plot) adjacent to each respiration collar. Forest floor (Oe + Oa) was collected from inside a 15 × 15 cm wooden frame after brushing the Oi litter aside from two locations within 1–2 m of each soil collar. We used 6 cm diameter tulip bulb corers to collect mineral soils from directly underneath the forest floor samples and from two additional locations per collar for the 0–3 cm and 3–10 cm mineral soil depth increments (*n* = 4 per collar). Soils were refrigerated at 4°C and processed in the lab 1–4 weeks after collection.

In the lab, soils were composited by collar location (to *n* = 5 per plot per depth), weighed, sieved (5.6 mm forest floor, 2 mm mineral soil), and subsampled for moisture determination and Rh_‐decomp_ measurements. Soils subsampled for Rh_‐decomp_ measurements were subsequently placed back into cold storage (4°C) before incubations. Bulk density was calculated for each sample from the total mass of sieved field‐moist soil and gravimetric soil water content measured on a 10 g subsample by drying at 105°C for 24 h.

For the Rh_‐decomp_ measurements, soils were incubated in the order that the stands were sampled and subsequently processed in the lab. We used 5 g of sieved forest floor material and 10 g of sieved mineral soil from 0 ‐ 3 cm and 3 ‐ 10 cm depths (5 samples per depth per plot) and placed them in 50 mL centrifuge tubes for incubation. Soils collected from all stands were initially incubated field‐moist within 2–6 weeks of collection, but below‐average precipitation in June and July of 2022 resulted in exceptionally dry soils at the time of collection for the last two stands. Therefore, we incubated soils from the last two stands a second time (10.5–12 weeks after collection) with water added to achieve the mean gravimetric soil water content as at the other four stands (see Supporting Information Methods for details).

We first pre‐incubated soils in a dark box for 24 h to reduce disturbance effects of subsampling. After the pre‐incubation, we flushed the headspace of the centrifuge tubes with CO_2_‐free air for 4 min at a rate of 120 mL/min to exchange headspace air approximately 10 times. Soils were then incubated for 24 h. Incubations were conducted in batches over ~11 weeks, ensuring that all plots (i.e., treatments) within a given stand experienced the same laboratory conditions. Further, all soils were incubated at least 15 days after field collection, and because root C released to the rhizosphere turns over rapidly (Kuzyakov et al. 2001; Kuzyakov 2002), measured fluxes should primarily consist of Rh_‐decomp_. We analyzed all gas samples for CO_2_ concentration and C isotopic composition at the Cornell Stable Isotope Laboratory, using a Thermo Scientific Delta V isotope ratio mass spectrometer, interfaced to a Thermo Scientific Gas Bench II. Rh_‐decomp_ fluxes were estimated from the total amount of headspace CO_2_ accumulation over the duration of the incubation period (see Supporting Information Methods for details). Carbon isotope data were used to check for leaks, enabled by the large difference in isotopic composition between atmospheric CO_2_ and Rh_‐decomp_. For all incubations, we estimated Rh_‐decomp_ C fluxes in three ways: first, in the commonly used units of per g dry soil mass (μg C g soil^−1^ h^−1^); second, when normalizing for differences in soil C as per g soil organic C (SOC; μg C g SOC^−1^ h^−1^); and third, on an areal basis (mg C m^−2^ h^−1^) accounting for differences in soil depth and bulk density measurements.

### 2.4 Soil Nitrogen Availability

We characterized inorganic soil nitrogen availability during the summer of 2019 using a buried resin bag technique (Allison et al. 2008). In late July 2019, we installed four resin bags in all plots, located 1–3 m toward the interior of the plot from each corner. Bags were placed 5 cm beneath the forest floor, when present, or 5 cm beneath the soil surface. Bags were removed from all plots 35–36 days after installation, rinsed with E‐pure water to remove debris, and frozen at –20°C until sent to the Cornell Nutrient Analysis Laboratory (CNAL) for extraction and colorimetric analyses of NO_3_ + NO_2_ and NH_4_ (see Supporting Information Methods for details). Though resin N measurements were collected 2–3 years before Rsoil and Rh_‐decomp_ measurements, they reflect treatment effects on soil N status accumulated over the long term (9 years). Subsequent N additions could further elevate soil N availability relative to measurements made in 2019, but our measurements capture the long‐term changes across plots and relative differences in soil N status that should drive soil respiration and decomposer activity responses.

### 2.5 Soil Physicochemical Measurements

We measured the pH of soils collected before treatment initiation (2009–2010), and again in 2019 and 2022. In 2009–2010, soils were collected at 4 locations per plot, using the same approach as in 2022 for the forest floor (15 × 15 cm blocks of Oe + Oa material), while mineral soils were sampled to 50 cm in 10 cm increments using a 9.6 cm diameter, diamond‐tipped rotary corer (Rau et al. 2011). Soils were dried and sieved (5.6 mm forest floor, 2 mm mineral soil), and pH was measured using an Accumet AB15 pH meter with a flushable junction probe (Fisher Scientific; Hampton, NH, USA) on a 1:2 soil:water slurry (10 g soil and 20 g DI water) for mineral soils and a 1:10 soil:water slurry for forest floors (Ross et al. 2015). In 2019, soils were collected from four locations per plot using 6 cm diameter corers to collect three cores per location for both forest floor (Oe + Oa) and surface mineral (0–10 cm) soils. Samples were composited in the field by depth within subplots. In the lab, soils were sieved field‐moist (5.6 mm forest floor, 4 mm mineral soil) and measured for pH as for 2009–2010. We also measured the pH of all field‐moist, sieved soils collected in 2022 used for Rh_‐decomp_ measurements using the same method. Though pH measurements are well‐replicated and should accurately capture pre‐existing and treatment‐driven differences in soil pH across plots, we note that differences in moisture content of samples measured pre‐treatment (dry) and post‐treatment (field‐moist) limit direct comparison of pH values between years (e.g., van Lierop and MacKenzie 1977).

Subsamples from all soils collected in 2022 were ground to a fine powder using a Kleco Ball Mill (Garcia Machine, Visalia, CA) and sent to the Cornell Stable Isotope Laboratory for analysis of soil C and N concentrations and isotopic composition using a Thermo Delta V isotope ratio mass spectrometer plumbed to a NC2500 Elemental Analyzer.

### 2.6 Statistical Methods

All data were analyzed using R version 4.4.1 (R Core Team 2024), and data and R code are available in the Environmental Data Initiative data repository (Frey et al. 2025). We fitted linear mixed‐effects models using the lme4 package (Bates et al. 2015) to test for treatment effects and to assess if the effects of treatments varied by stand age (i.e., primary vs. secondary). Models for all response variables included fixed parameters for the effects of sites, treatments, stand ages, and the interactive effects of stand age and treatments. For all variables with multiple within‐plot measurements (soil pH, resin N availability, soil Rh_‐decomp_ fluxes, and monthly Rsoil measurements), models contained two random intercept terms that allowed intercepts to vary by stands (*N* = 6) and plots within stands (*N* = 24). For variables with only one plot‐level measurement (annual Rsoil), we only included a random effect term for stand (see Supporting Information Methods for additional details).

We used the lmerTest package (Kuznetsova et al. 2017) to calculate type III *F*‐tests to assess if interaction terms were significant. *F*‐tests for treatment × stand age interactions were not significant in any models, so we used the emmeans package (Lenth 2024) to estimate overall treatment effects across all stands by comparing differences in estimated marginal means. For the monthly Rsoil model (see Supporting Information Methods for details), we also compared differences in estimated marginal means between treatments within months. We used Tukey corrections to adjust *p*‐values for familywise error rates for comparisons between treatments. When effects of both N or both acidification treatments were directionally consistent relative to the control, we made additional comparisons between estimated marginal means for +N or +acidification treatments and controls. For these tests, we used multivariate *t* (mvt) corrections to adjust *p*‐values when comparing overall +N and overall +acidification effects to controls.

In addition to treatment effects, we examined relationships between measures of soil N availability, soil pH, Rh_‐decomp_, and annual Rsoil using linear mixed‐effects models (see Supporting Information Methods for details). To test the relationship between Rsoil and Rh_‐decomp_ fluxes, we summed areal estimates of Rh_‐decomp_ fluxes across all three depths and averaged fluxes by plot. We assessed the statistical significance of predictors using Type III *F*‐tests (lmerTest package), and we estimated marginal *R* ^2^ for each predictor using the glmm.hp package (Lai et al. 2022), enabling us to assess each variable's contribution to the total variance explained by the fixed effects.

Finally, we investigated if the relationship between Rh_‐decomp_ and Rsoil differed in plots fertilized with N versus those that did not receive fertilizer, and used this approach to identify treatment effects on R_‐root‐assoc_. To that end, we used a linear mixed‐effects model with categorical treatments and areal‐based Rh_‐decomp_ as interactive fixed effects, with site as an additive fixed effect and stand as a random effect to predict annual Rsoil. A type III *F*‐test demonstrated that the interaction was not significant (*p* = 0.78), so we refit an additive model with the same predictors and response. This additive model enables partitioning Rh_‐decomp_ and R_‐root‐assoc_ responses to treatments (Equations (4), (5), (6)). Conceptually, the plant‐driven soil CO_2_ efflux represented here as R_‐root‐assoc_ (i.e., the sum of Ra_‐root_ + Rh_‐myco_ + Rh_‐rhizo_, Equation 2) can be substituted into Equation (1) to yield Equation (4):

(4) $$\text{Rsoil}={\mathrm{Rh}}_{-\text{decomp}}+R_{-\text{root}-\text{assoc}}$$

A simplified form of the linear relationship between Rsoil and Rh_‐decomp_ included in our regression model is:

(5) y=mx+b,

where *m* defines the effect of a one unit increase in Rh_‐decomp_ (*x*, here in mg C m^−2^ h^−1^) on Rsoil (*y*, Mg C ha^−1^ year^−1^), and b should reflect the contribution of R_‐root‐assoc_ to Rsoil (also in Mg C ha^−1^ year^−1^). Together, Equations (4) and (5) demonstrate that treatment‐level differences in the regression model's intercept (b, or R_‐root‐assoc_) reflect differences in R_‐root‐assoc_ by treatment (Equation 6), enabling us to assess how treatments impacted the plant‐driven R_‐root‐assoc_ component of Rsoil.

(6) $$\text{Rsoil}=m\left({\mathrm{Rh}}_{-\text{decomp}}\right)+R_{-\text{root}-\text{assoc}}$$

## 3 Results

Soil pH and N availability, heterotrophic respiration, and soil respiration all responded to our experimental treatments, as described further below. Several properties varied by site, but stand age had little effect overall or in mediating the treatment effects of primary concern and is not discussed further (Tables S1–S4).

### 3.1 Soil Chemistry

**FIGURE 1:**
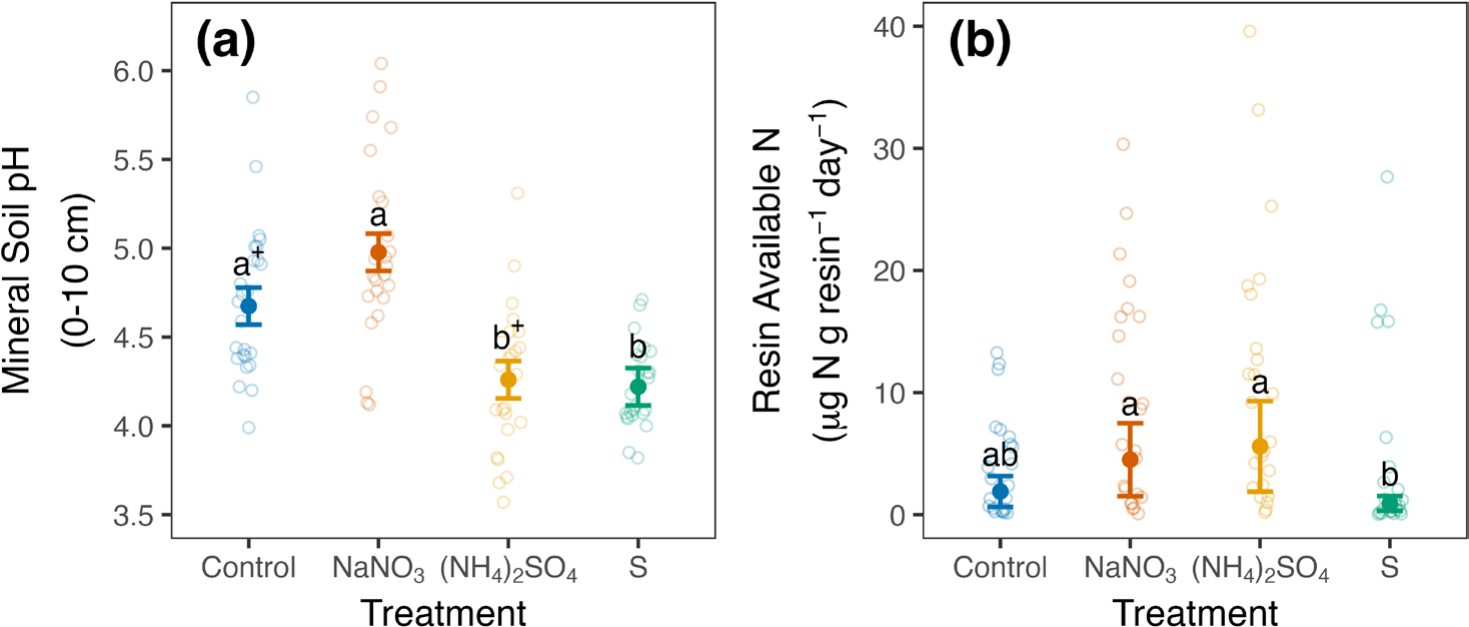
Surface mineral soil pH (a) and resin available nitrogen (b) by treatment, after 8 and 9 years of treatment, respectively. Solid points are estimated marginal means (a) or back‐transformed estimated marginal means (b) and error bars are associated standard errors estimated from linear mixed‐effects models. Hollow points are measured values. Differences between treatments are statistically significant (*p* ≤ 0.05) when treatments do not share common letters. When treatments do not share common letters and have common + symbols, 0.1 ≥ *p* > 0.05. Treatments are designated on the *x*‐axis as: (1) control = no fertilization; (2) NaNO_3_ = sodium nitrate addition; (3) (NH_4_)_2_SO_4_ = ammonium sulfate addition; and (4) S = elemental sulfur addition.

Surface mineral soil (0–10 cm) pH did not differ between treatments before the initiation of fertilizer additions (Figure S2), but showed marked differences by 2019 after 8 years of treatments (Tables S1 and S5). Both acidification treatments led to large reductions in soil pH relative to the control (Figure 1a; S vs. control, −0.45 pH units, *p* = 0.04; (NH_4_)_2_SO_4_ vs. control, −0.41 units, *p* = 0.07). The deacidifying nitrogen treatment had a significantly higher pH than the acidification treatments (Figure 1a; NaNO_3_ vs. S, +0.76 units, *p* = 0.001; NaNO_3_ vs. (NH_4_)_2_SO_4_, +0.72 units, *p* = 0.002), and had the highest pH values on average but did not differ significantly from control plots (NaNO_3_ vs. control, +0.30 units, *p* = 0.22).

Median resin‐available N was 2.6‐fold higher in N‐treated plots than in control plots on average (Table S5; +N vs. control, *p* = 0.02), but it was highly variable, particularly in N‐treated plots (Figure 1b; (NH_4_)_2_SO_4_ vs. control, *p* = 0.11; NaNO_3_ vs. control, *p* = 0.24). N availability in the N‐treated plots was 5–6‐fold higher than in the acidification‐only treatment ((NH_4_)_2_SO_4_ vs. S, *p* = 0.006; NaNO_3_ vs. S, *p* = 0.01), which was lowest on average but did not differ significantly from the control plots (*p* = 0.37).

### 3.2 Heterotrophic Respiration (Rh_‐decomp_)

**FIGURE 2:**
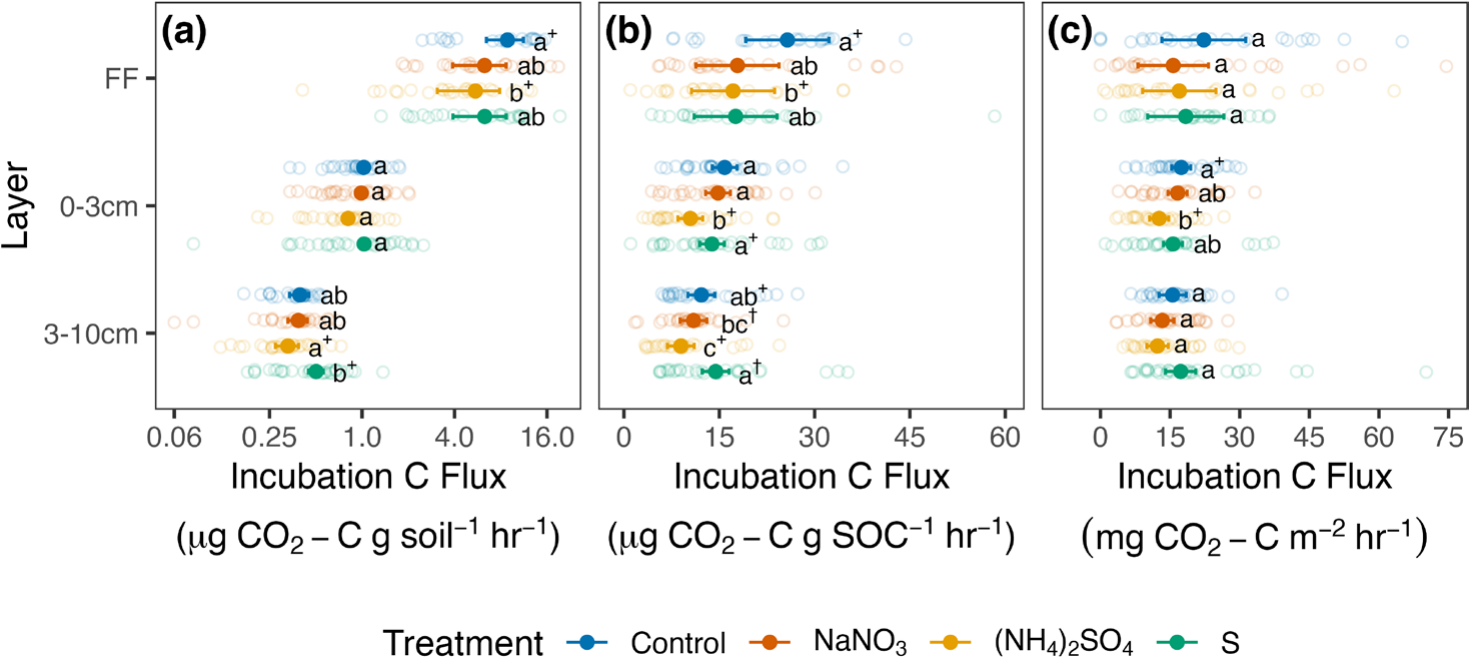
Forest floor (FF), 0–3 cm mineral soil, and 3–10 cm mineral soil incubation C fluxes per gram soil (a; Rh_‐decomp/mass_), per g soil organic carbon (SOC) (b; Rh_‐decomp/SOC_), and per m^2^ (c; Rh_‐decomp/area_), from incubations with water added substituted for field moist soils in two stands where soils were particularly dry. Solid points are estimated marginal means or back‐transformed estimated marginal means when response variables were transformed to meet model assumptions. Error bars are associated standard errors. Hollow points are measured fluxes. Compact letters designate statistically significant (*p* ≤ 0.05) differences between treatments within each depth separately, not differences between depths. Differences between treatments are statistically significant when treatments do not share common letters. When treatments do not share common letters and share + or † symbols, 0.1 ≥ *p* > 0.05.

We expected that acidifying and deacidifying N additions might exert differing effects on Rh_‐decomp_, but both N treatments tended to suppress Rh_‐decomp_ per unit soil mass (Rh_‐decomp/mass_). N fertilization effects were largest in the forest floor, where N additions suppressed Rh_‐decomp/mass_ by 34% overall (Table S6; +N vs. control, *p* = 0.02), and both forms of N addition tended to suppress Rh_‐decomp/mass_ (Figure 2a; (NH_4_)_2_SO_4_ vs. control, −38%, *p* = 0.06; NaNO_3_ vs. control, −29%, *p* = 0.17). In contrast, N additions did not detectably affect Rh_‐decomp/mass_ at either mineral soil depth (Figure 2a). Acidification alone did not significantly affect Rh_‐decomp/mass_ relative to the control, but effects varied directionally by soil depth. As predicted, acidification alone tended to suppress Rh_‐decomp/mass_ in the forest floor (Figure 2a; S vs. control, −29%, *p* = 0.18), and Rh_‐decomp/mass_ was 33% lower than controls across both acidification treatments (Table S6; −pH vs. control, *p* = 0.02). Surprisingly, in the 3–10 cm mineral soil, instead of suppressing Rh_‐decomp/mass_, acidification alone tended to stimulate it, though not significantly (S vs. control, +28%, *p* = 0.35). These responses led to a 42% difference in Rh_‐decomp/mass_ between the two acidification treatments in 3–10 cm soils (*p* = 0.07), with marked suppression of Rh_‐decomp/mass_ by combined N and acidification compared to acidification alone.

Rh responses per unit soil organic C (Rh_‐decomp/SOC_) were generally consistent with responses per unit mass in the forest floor (Table S7), where N additions and acidification treatments both suppressed Rh_‐decomp/SOC_ (Figure 2b; +N vs. control, −32%, *p* = 0.02; −pH vs. control, −32%, *p* = 0.02), and all three of the individual treatments tended to suppress Rh_‐decomp/SOC_ by a similar amount (NaNO_3_ vs. control, −30%, *p* = 0.13; (NH_4_)_2_SO_4_ vs. control, −33%, *p* = 0.09; S vs. control, −32%, *p* = 0.11). In the 0–3 cm depth, treatment effects on Rh_‐decomp_ emerged when normalized per unit SOC, while none were detected for Rh per unit soil mass. N fertilization suppressed Rh_‐decomp/SOC_ by 21% (Table S7; *p* = 0.02) and acidification suppressed Rh_‐decomp/SOC_ by 24% (Table S7; *p* = 0.009). However, in contrast with the forest floor where all treatments exerted similar effects on Rh_‐decomp/SOC_, in the 0–3 cm soils, the acidifying N treatment caused substantial suppression (−35%, *p* = 0.005), while the other treatments had smaller, non‐significant reductions in Rh_‐decomp/SOC_ (NaNO_3_ vs. control −7%, *p* = 0.83; S vs. control −13%, *p* = 0.42). Similar to the 0–3 cm soils, in 3–10 cm mineral soils, N addition reduced Rh_‐decomp/SOC_ (Table S7; +N vs. control, −18%, *p* = 0.051), driven primarily by the acidifying N treatment ((NH_4_)_2_SO_4_ vs. control, −27%, *p* = 0.08). As for the Rh_‐decomp/mass_ results, the acidification‐only treatment tended to stimulate Rh_‐decomp/SOC_, with fluxes not significantly greater than in controls (S vs. control, +18%, *p* = 0.29), but much greater than the acidifying N treatment (S vs. (NH_4_)_2_SO_4_, 47% difference, *p* = 0.003).

**FIGURE 3:**
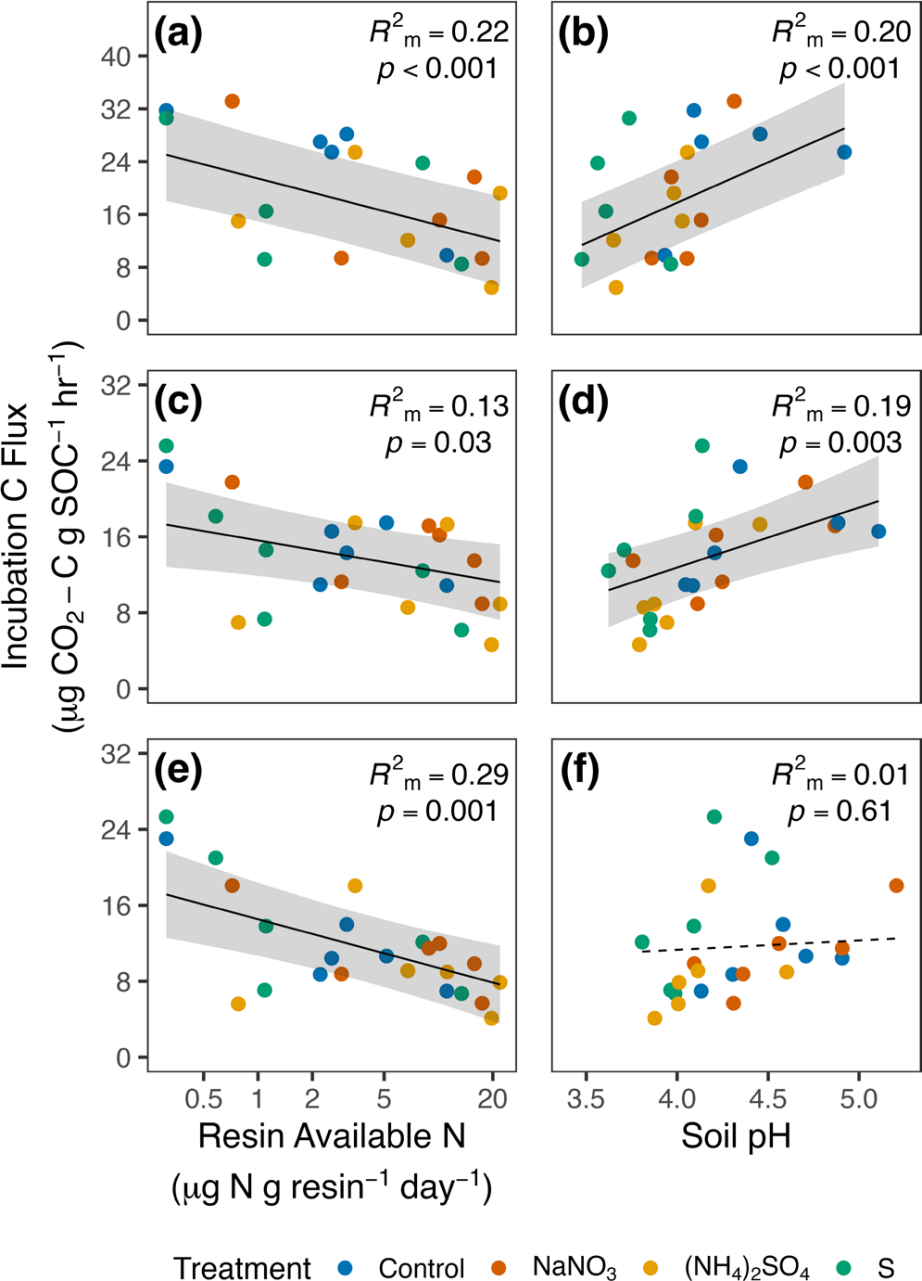
Relationships between incubation C fluxes (Rh_‐decomp/SOC_) and resin available N and soil pH in the forest floor (a, b), 0–3 cm mineral soil (c, d), and 3–10 cm mineral soil (e, f). Points are plot‐level means for both variables, solid black lines represent predicted values from linear mixed‐effects models when predictors are statistically significant (*p* ≤ 0.05), and dashed lines show predicted relationships for non‐significant predictors. Gray ribbons display 95% confidence intervals for predicted values. R^2^_m_ is the marginal *R* ^2^ for each variable.

We capitalized on large variation in soil N availability and pH across plots to further evaluate how these factors impacted Rh_‐decomp/SOC_. In both the forest floor and 0–3 cm mineral soils, N availability and pH were important predictors of Rh_‐decomp/SOC_ (Figure 3a–d; Table S8). At both depths, Rh_‐decomp/SOC_ decreased with increasing soil N availability (Forest Floor, *F*_1,14.2_ = 21.8, *p* < 0.001; marginal *R* ^2^ = 0.22; 0–3 cm mineral soil, *F*_1,18.9_ = 5.7, *p* = 0.03; marginal *R* ^2^ = 0.13), but increased with increasing soil pH (Forest Floor, *F*_1,13.1_ = 60.0, *p* < 0.001; marginal *R* ^2^ = 0.20; 0–3 cm mineral soil, *F*_1,17.3_ = 12.4, *p* = 0.003; marginal *R* ^2^ = 0.19). In contrast, soil pH did not affect Rh_‐decomp/SOC_ in 3–10 cm mineral soils (Figure 3f; Table S8; *F*_1,17.3_ = 0.27, *p* = 0.61; marginal *R* ^2^ = 0.01), but N availability was again an important predictor of Rh_‐decomp/SOC_ at this depth (Figure 3e; *F*_1,18.7_ = 13.8, *p* = 0.001; marginal *R* ^2^ = 0.29).

Areal Rh_‐decomp_ estimates (Rh_‐decomp/area_) were more variable than those per unit soil mass or SOC, likely due to additional uncertainty associated with soil bulk density measurements needed to estimate fluxes per unit area; however, treatment effects were generally directionally consistent using all Rh metrics (Figure 2a–c; Table S9). Acidification effects of suppressing Rh_‐decomp/area_ in the forest floor were partly offset by stimulation in 3–10 cm mineral soils, while N tended to either suppress or exert neutral effects on Rh_‐decomp/area_ across all layers. Together, these results illustrate that both soil acidity and soil N availability are important controls of Rh_‐decomp_, but that their separate effects vary with soil depth.

For the two stands with especially dry soils at the time of collection, median Rh_‐decomp_ fluxes from incubations with added water were approximately 72% higher than those incubated field‐moist. However, reanalyses of treatment effects on Rh_‐decomp_ fluxes using values from field‐moist rather than rewet soils led to directionally consistent treatment effects compared to those observed in the rewet soils (Figure S3; Tables S10–S12).

### 3.3 Soil Respiration

**FIGURE 4:**
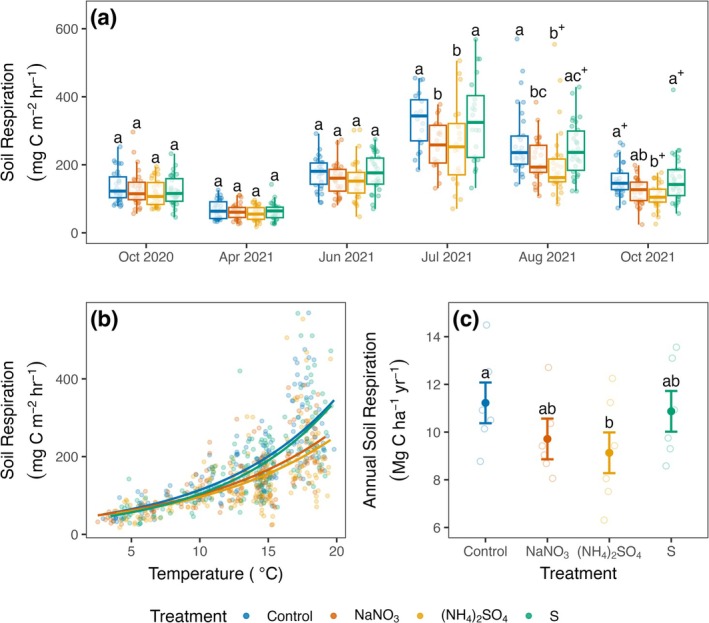
Measured soil respiration fluxes by treatment from October 2020 to October 2021 (years 10–11 of treatment) (a), measured soil respiration flux versus soil temperature (10 cm depth) (b), and estimated annual soil respiration by treatment (c). Bars in boxplots in panel (a) are medians. Points in panels (a) and (b) are collar‐level measured respiration fluxes, and curves in panel (b) show treatment level relationships between soil respiration and temperature, using the equation Rsoil = a*e^bt^, where Rsoil = soil respiration flux, a and b are fitted parameters, and *t* = soil temperature (°C) at 10 cm depth. Solid points in panel (c) are estimated marginal means and error bars are associated standard errors. Hollow points are plot‐level annual Rsoil estimates. Differences between treatments are statistically significant (*p* ≤ 0.05) when treatments do not share common letters. When treatments do not share common letters and share + symbols, 0.1 ≥ *p* > 0.05. In panel (a), letters compare differences within each month, but not between months.

Acidifying N additions reduced annual Rsoil substantially relative to controls (−19%; −2.1 Mg C ha^−1^ year^−1^, *p* = 0.048), driven by lower fluxes in the mid‐ to late‐growing season in 2021 (Figure 4; Table S13). The deacidifying N treatment also trended toward suppression of Rsoil (−13%, −1.5 Mg C ha^−1^ year^−1^, *p* = 0.19). Collectively, fluxes across all N‐treated plots were 1.8 ± 0.6 Mg C ha^−1^ year^−1^ lower than in control plots (Table S14; *p* = 0.02, 95% CI [0.3, 3.3]). In contrast with N additions, the acidification‐only treatment had no effect on respiration annually (*p* = 0.96) or in any of the months measured (Figure 4; Tables S13–S14).

**FIGURE 5:**
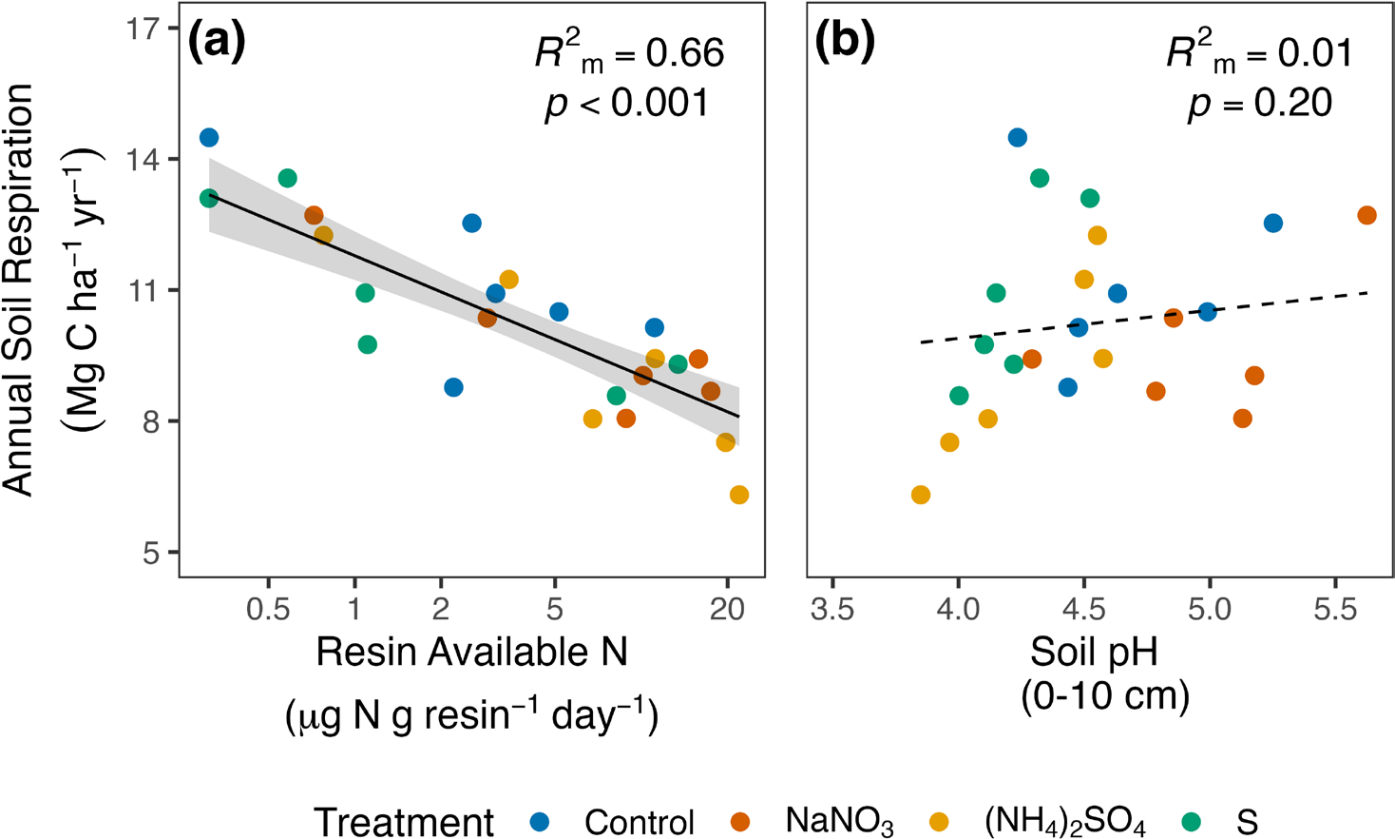
Relationships between estimated annual soil respiration (Rsoil) from Figure 4 and resin available N (a) and soil pH (b) from Figure 1. Points are plot‐level means for predictors and plot‐level estimates for Rsoil, solid and dashed black lines represent predicted values from linear mixed‐effects models when predictors are statistically significant (*p* ≤ 0.05) or non‐significant, respectively, and gray ribbons display 95% confidence intervals for predicted values. *R* ^2^_m_ is the marginal *R* ^2^ for each variable.

Examination of drivers of Rsoil across plots revealed that soil N availability was a strong predictor of annual Rsoil, with Rsoil decreasing sharply with increasing soil N availability (Figure 5a; Table S15; *F*_1,19_ = 58.7, *p* < 0.001; marginal *R* ^2^ = 0.66). In contrast, soil pH did not explain variation in Rsoil across all plots (Figure 5b; Table S15; *F*_1,19_ = 1.8, *p* = 0.20; marginal *R* ^2^ = 0.01), though Rsoil tended to be lowest in the most acidic plots.

**FIGURE 6:**
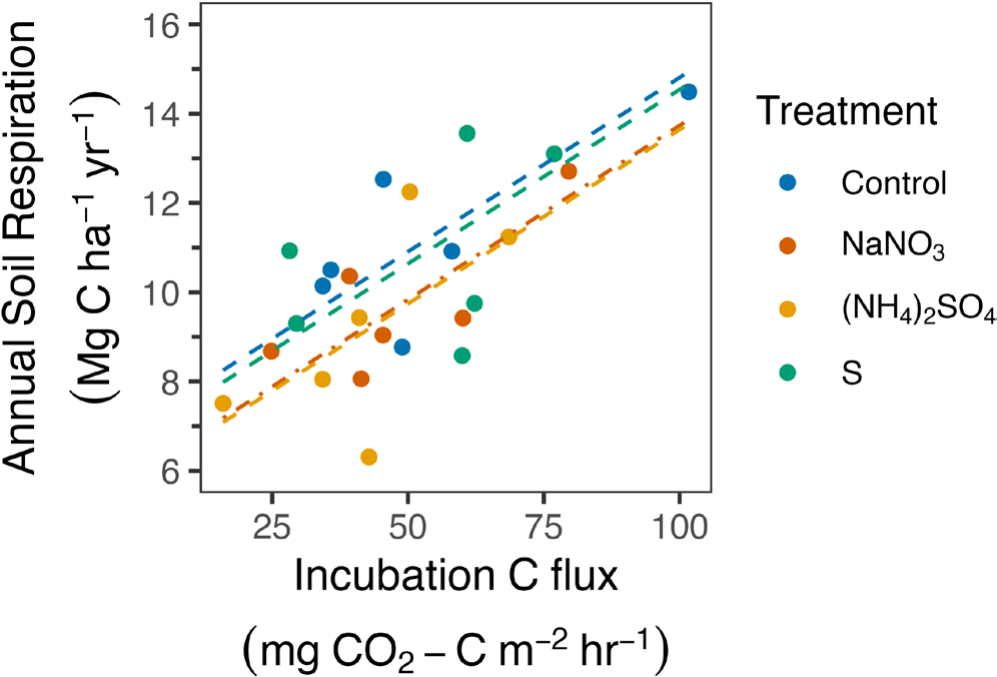
Relationships between estimated annual soil respiration (Rsoil) and incubation C fluxes (${\mathrm{Rh}}_{-\text{decomp/area}}$) by treatment. Points are plot‐level means for incubation C fluxes and plot‐level estimates for Rsoil and dashed lines represent predicted values from a linear mixed‐effects model. Dash types differ between the NaNO_3_ and (NH_4_)_2_SO_4_ treatments for visualization purposes only.

Rsoil correlated strongly with Rh_‐decomp/area_ summed across all three surface soil layers (Table S16; *F*_1,13.3_ = 35.2, *p* < 0.001; marginal *R* ^2^ = 0.54), suggesting that Rh_‐decomp_ values explained a large fraction of the variability in Rsoil. However, N fertilization modified this relationship, reducing Rsoil relative to Rh_‐decomp_ by 1.12 ± 0.44 Mg C ha^−1^ year^−1^ when compared with controls (Figure 6; Tables S14 and S17; +N vs. control, *p* = 0.03, 95% CI = [0.09, 2.15]). This reduction demonstrates that N additions decreased Rsoil after accounting for the role of Rh_‐decomp_ and provides a means of quantifying the effects of N in suppressing R_‐root‐assoc_ (Equations (4), (5), (6)).

## 4 Discussion

### 4.1 Whole‐Ecosystem Soil Respiration Suppressed by Nitrogen, Not pH

By independently manipulating N availability and soil pH, we sought to disentangle long‐confounded and theoretical expectations about the role of acidification in driving Rsoil responses to N addition (e.g., Janssens et al. 2010; Averill and Waring 2018). Our findings reveal that in these mixed temperate deciduous forests, soil acidification alone had no overall effect on Rsoil, whereas N addition reduced the flux by 13 to 19% (Figure 4; 1.5–2.1 Mg C ha^−1^ year^−1^). This suppression by N is substantial compared with Rsoil responses reported in meta‐analyses of the effects of N addition in forests (i.e., 10%, Janssens et al. 2010; 1.4%, Zhou et al. 2014). Further, compared across plots, annual Rsoil decreased sharply with increasing soil N availability. Soil pH had no significant overall effect on the flux, though Rsoil tended to be lowest in the most acidic plots, and acidification could play a larger role in more acidic conditions. Together, these results demonstrate the overriding role of N availability as a control over Rsoil in these forests.

Our annual Rsoil estimates (11.2 ± 0.8 Mg C ha^−1^ year^−1^ in control plots) are comparable to those recently reported from similar forests in the same region (~9.5–12 Mg C ha^−1^ year^−1^ in Garvey et al. 2022; Reinmann and Templer 2018; Smith et al. 2019), but greater than most earlier estimates for similar forests (~6.5–9 Mg C ha^−1^ year^−1^ in Bae et al. 2015; Fahey et al. 2005; Fisk et al. 2004; and Giasson et al. 2013). Global change factors including rising temperatures (Bond‐Lamberty and Thomson 2010; Lei et al. 2021) and atmospheric CO_2_ concentrations (Akande et al. 2023; King et al. 2004) are expected to exacerbate N limitation and stimulate Rsoil (e.g., Mason et al. 2022), and may explain the relatively high annual Rsoil rates in control plots in the present study relative to earlier estimates from the same region. These same factors might also contribute to the large responses to N addition that we observed.

Our results differ from some other studies that have reported a prominent role of soil pH change in driving suppression of Rsoil. Several factors could contribute to these contrasting observations. First, meta‐analyses have shown that acidification can suppress Rsoil (Meng et al. 2019; Lu et al. 2022), but only across very wide soil pH ranges (pH 3–8). Moreover, acidification treatments applied to sites that are more alkaline can reduce pH to a much greater degree (Meng et al. 2019), and larger declines in soil pH more strongly suppress Rsoil (Lu et al. 2022). Though our experimental acidification treatments did reduce soil pH (Figure 1a, 0.41–0.45 units), Meng et al. (2019) and Lu et al. (2022) both show shallow relationships between the magnitude of experimental soil acidification and Rsoil responses, suggesting that stronger soil acidification than induced by our treatments may be necessary to detect effects on Rsoil. Still, our treatments do reflect a 2.5 to 2.8‐fold increase in hydrogen ion concentration in already acidic soils. Rsoil responses to acidification may also be sensitive to initial soil pH. For example, a study in the Czech Republic found that Rsoil was suppressed by acidification, but not N, after three years of similar N and S application rates as our study added to an acidic (pH 3.83) spruce stand (Oulehle et al. 2018). However, a paired study in a less acidic beech stand (pH 4.34) showed no Rsoil responses to either acidification or N additions. Notably, the direct effect of enhanced free acidity in already very acidic soils could suppress the activity of extracellular enzymes produced by microbial decomposers (Shi et al. 2023; Sinsabaugh 2010), and increase toxic metal availability, which can damage fine roots and reduce their growth (Cronan et al. 1989; Cronan and Grigal 1995; Godbold et al. 1988). Such effects could elicit declines in Rh_‐decomp_ and R_‐root‐assoc_, respectively, ultimately reducing Rsoil.

### 4.2 Experimental Scale and Rsoil Responses to Treatments in Forests

One of the primary differences between our study and the few others that have evaluated how pH and N availability affect Rsoil is that we applied treatments at the ecosystem scale and for a long duration (~10 years). Spatial scale may be an important factor when designing studies that investigate belowground C cycling processes in forests, due to the large lateral extent of tree roots (Lyford and Wilson 1964; Meinen et al. 2009). Our large plots with 10 m wide treated buffer areas (total treated area = 3600 m^2^ per plot) were designed to fertilize the majority of root systems of most of the trees and thus capture both autotrophic and heterotrophic responses to N addition and acidification. By contrast, the other forest experiments (Li et al. 2018; Oulehle et al. 2018) applied treatments at smaller scales (9–100 m^2^) that would not elicit representative responses of R_‐root‐assoc_ because most of the tree root systems would lie outside the treated areas. Exactly how this bias would affect Rsoil responses to acidification and N treatments is uncertain, but higher ecosystem‐level N availability can result in lower plant belowground C allocation in similar forests (Bae et al. 2015; Eastman et al. 2021), consistent with our finding that N additions suppressed R_‐root‐assoc_. In contrast, because roots and mycorrhizal hyphae can proliferate in small, localized patches with higher nutrient availability (Chen, Koide, et al. 2016; Hodge 2004, 2006), small plots that only partially fertilize root systems might instead increase R_‐root‐assoc_. Similarly, because acidification can decrease soil nutrient availability over the long term (Bailey et al. 2005; Driscoll et al. 2001), trees in large, acidified plots may increase C allocation to roots and the rhizosphere, whereas similar conditions in small plots might encourage root redistribution to more favorable microsites outside plots. Combined with suppression of soil heterotrophic activity, acidification could then reduce Rsoil in small plots, but not large plots. Hence, our finding that increased N availability, rather than acidification, suppresses Rsoil in these temperate forests may conflict with other studies due to the spatial scale of the experimental manipulations and the ability of our large‐scale treatments to capture the response of plant‐driven respiration fluxes.

### 4.3 Heterotrophic Respiration (Rh_‐decomp_) Suppressed by Increased N Availability and Soil Acidification, But Acidification Effects Vary by Soil Depth

We expected that acidification would suppress Rh_‐decomp_ by decreasing microbial activity, and that increased N availability without acidification might stimulate the flux by increasing soil saprotrophic activity, especially in C‐rich surface horizons, as predicted by the Carbon Acidity Mineral Protection (CAMP) framework (Averill and Waring 2018). We found that in the forest floor, acidification did suppress Rh_‐decomp/SOC_ overall (−32%), but so did N additions (−32%), with a similar magnitude of suppression by all three treatments. Surprisingly, this suppression included the deacidifying N treatment, which contrasts with expectations of the CAMP framework. In mineral soil, both forms of N addition showed directionally negative or neutral effects on Rh_‐decomp_, while the acidification‐only treatment showed neutral or even positive effects. Therefore, when totaled over all measured soil depths, increased N tended to cause net suppression of Rh_‐decomp_, whereas suppression by acidification in the forest floor was partially offset by stimulation at depth (Figure 2). Both of these findings are not only inconsistent with the CAMP framework but sometimes opposite to expectations.

Our results showing that N additions tended to suppress Rh_‐decomp_ even in de‐acidifying treatments do not support the hypothesis that microbial N limitation acts as a constraint on soil heterotrophic activity. N‐induced suppression of decomposition is often greatest in the forest floor (Frey et al. 2014; Liu and Greaver 2010), where relatively high C:N ratios might produce stronger microbial N limitation relative to typically lower C:N ratios in mineral soils, due to a larger stoichiometric mismatch between microbes and organic matter in the forest floor than in the mineral soil. In our study, the magnitude of Rh_‐decomp_ suppression from N additions was largest in the forest floor, demonstrating that even where microbial N limitation might be greatest, N addition did not release heterotrophic activity from nutrient limitation in ways that result in greater C mineralization. Notably, forest floor C:N ratios at these mixed deciduous sites are relatively low (< 21 on average), such that the N supplied through its decomposition should generally suffice to meet microbial demands. Hence, our findings are consistent with other studies in temperate forests where N additions suppress, rather than stimulate, microbial activity, leading to soil C accumulation (Bowden et al. 2019; Frey et al. 2014; Lovett et al. 2013). These effects probably owe to changes in microbial community composition and function, where increasing soil N availability often reduces fungal production of enzymes that degrade lignin (e.g., Carreiro et al. 2000; DeForest et al. 2004; Frey et al. 2014). Such an effect likely explains the tendency for N additions to suppress Rh_‐decomp_ in our study.

We expected that acidification would decrease Rh_‐decomp_ across all soil layers because acidic soils typically have lower microbial activity and biomass than less‐acidic soils (Anderson and Domsch 1993; Bååth and Anderson 2003; Chen, Li, et al. 2016; Francis 1982). This suppression did indeed occur in the forest floor, but surprisingly, acidification alone tended to increase Rh_‐decomp_ in 3–10 cm mineral soils, while the acidifying N treatment tended to suppress Rh_‐decomp_ in all layers (Figure 2). The suppression of mineral soil Rh_‐decomp_ in plots receiving acidifying N additions may manifest from the suppression of oxidative enzyme activities (Carreiro et al. 2000; DeForest et al. 2004; Frey et al. 2014) or reduced detrital C inputs from fine roots (Dong et al. 2022; Peng et al. 2017). By contrast, long‐term acidification may be inducing N limitation, which could spur greater fine root production. However, because differences between the acidification‐only and acidifying N treatment persisted after standardizing fluxes for soil C content, it is unlikely that changes in the concentration of detrital root C available for decomposition explain this response. Though the mechanism underpinning differing Rh_‐decomp_ responses to acidification with or without N fertilization in this study is unclear, our results do not support the notion that soil acidification is the primary driver of decreased Rh_‐decomp_ under N enrichment in these temperate forest mineral soils, but that N as a nutrient also drives Rh_‐decomp_ suppression.

### 4.4 Partitioning N and pH Effects on Plant‐ and Decomposition‐Driven Rsoil Responses

Responses of Rsoil to changes in soil N availability and pH reflect net effects on Rh_‐decomp_, Ra_‐root_, Rh_‐myco_, and Rh_‐rhizo_. However, these terms are exceedingly difficult to partition at the whole‐ecosystem scale and are subject to important experimental artifacts. Our study design allowed us to estimate how changes in root‐associated respiration (R_‐root‐assoc_) fluxes (Ra_‐root_, Rh_‐myco_, and Rh_‐rhizo_) affected Rsoil responses to N additions and soil acidification by examining treatment‐level offsets in the relationship between Rsoil and Rh_‐decomp_ (Equations (4), (5), (6)). Our analysis indicated that the two N treatments suppressed R_‐root‐assoc_ by 1.12 Mg C ha^−1^ year^−1^ (Figure 6), which amounts to 62% of the overall effect of N addition on Rsoil (−1.8 Mg C ha^−1^ year^−1^). By difference, suppression of Rh_‐decomp_ by N at the annual scale would be ~0.7 Mg C ha^−1^ year^−1^. This R_‐root‐assoc_ response is nearly identical to the reduction of belowground C allocation (1.14 Mg C ha^−1^ year^−1^) reported in Eastman et al. (2021), estimated using a mass balance approach in a watershed‐scale (NH_4_)_2_SO_4_ addition to a temperate deciduous forest.

Our partitioning approach allows estimation of treatment effects on the plant‐ and decomposition‐driven processes that contribute to Rsoil responses. The laboratory incubations used in this study to assess Rh_‐decomp_ may not reflect in situ Rh_‐decomp_, largely because they are short‐term assays of highly disturbed soils. Nevertheless, they do provide a common index of CO_2_ generated by decomposition in the absence of plants, and these sorts of incubations are commonly used for this purpose (Billings and Ziegler 2008; Cleveland and Townsend 2006; Kutsch et al. 2010). Importantly, as is the case for in situ approaches for partitioning components of Rsoil such as trenching and girdling, our approach does not allow separation of Ra_‐root_ from the other plant‐associated fluxes or priming‐driven Rh_‐decomp_.

Our findings demonstrate that changes in belowground C allocation that drive R_‐root‐assoc_ play an important role in how Rsoil responds to N addition. Further, our approach may actually underestimate the effect of shifts in plant C allocation on Rsoil by failing to fully capture effects of Rh_‐rhizo_ on Rh_‐decomp_ through priming of soil organic matter (SOM) decomposition (Dijkstra and Cheng 2007). Decreased rhizosphere C allocation by plants to microbes could reduce Rsoil not only through the direct contribution of the Rh_‐rhizo_ flux to Rsoil but also through its effects on stimulating SOM mineralization (Rh_‐decomp_) (Finzi et al. 2015; Phillips and Fahey 2007). Our short‐term incubations could have captured some effects of recent belowground C inputs on Rh_‐decomp_, but root exudates utilized by rhizosphere microbes are probably largely consumed within days of their production (Kuzyakov et al. 2001). Therefore, our incubations, conducted ~2–11 weeks after soil collection, should have mostly captured Rh_‐decomp_ rather than direct contributions to Rh from Rh_‐rhizo_ or, consequently, effects of Rh_‐rhizo_ on Rh_‐decomp_ as mediated by priming.

The soils examined in this study are more acidic than the optimum values expected for large positive priming effects (pH 5.5–7.5), but positive priming does still occur within the pH range of soils at our sites (Wang and Kuzyakov 2024). Priming effects on Rh_‐decomp_ can be substantial, and if changes in rhizosphere C flux impacted SOM priming in our study, they might play an important role in Rsoil responses to N addition. For example, Finzi et al. (2015) demonstrated that rhizosphere effects in temperate forests can be responsible for up to one‐third of total C mineralized in soils, and that these effects can manifest even from a fairly small C contribution by plants to the rhizosphere (e.g., 6% of NPP, Finzi et al. 2015). Hence, even a small reduction in belowground C allocation to the rhizosphere in N‐treated plots could have caused considerable suppression of in situ Rh_‐decomp_. Though relatively underexplored in forest ecosystems, several studies have demonstrated that N fertilization can impact rhizosphere C cycling (Phillips and Fahey 2007; Shan et al. 2018), and further investigation into these effects could provide valuable insights into how increased N availability alters soil C dynamics.

### 4.5 Implications: Ongoing and Future N Limitation Should Stimulate Rsoil

Few experiments have been conducted to test how pH and N independently or interactively affect Rsoil and its components in forests. To our knowledge, this is the first study in forest ecosystems using large, long‐term plots where fertilization of whole root systems enables assessment of both plant‐ and microbially driven responses to changes in soil N availability and pH. Overall, our results challenge the notion that soil acidification drives Rsoil suppression under soil N enrichment. Acidification alone did decrease Rh_‐decomp_ in the forest floor, but that effect was partially offset by higher fluxes in 3–10 cm mineral soils, and acidification alone had no effect on R_‐root‐assoc_. Together, these responses resulted in no acidification effect on the total Rsoil flux. Instead, we find that in these temperate forests, increased soil N availability, not soil acidification, causes Rsoil suppression. Rsoil responses to N were driven by reductions in both Rh_‐decomp_ and R_‐root‐assoc_, constituting roughly 1/3 and 2/3 of the decrease, respectively. It is possible that acidification may play a stronger role in suppressing Rh_‐decomp_ over longer timescales or in more acidic conditions.

Our findings have important implications for how various global change factors will impact C fluxes in temperate forest ecosystems. Both N and S deposition have decreased substantially in North America and Europe over the past two decades (Aas et al. 2019; Ackerman et al. 2019), and though soils in Europe have been slow to deacidify (Johnson et al. 2018), eastern North American soils are showing many signs of recovery (Lawrence et al. 2015). Our results suggest that both decreased N deposition and soil deacidification should lead to increased decomposition (Rh_‐decomp_), which could reduce forest soil C storage. However, decreased N deposition will have more pronounced impacts than soil deacidification on Rsoil, especially when coupled with rising atmospheric CO_2_ levels that may increase plant N demand and N sequestration in organic matter pools with slow turnover times (Luo et al. 2004; Norby et al. 2010). Both of these factors can reduce soil N availability, making them potential drivers of observed N oligotrophication in many terrestrial ecosystems (Groffman et al. 2018; Mason et al. 2022). Our findings indicate that greater N limitation will increase Rsoil by both plant‐ and microbially mediated mechanisms, but especially the plant‐driven fluxes. First, trees may allocate more C belowground to access increasingly scarce resources as N availability decreases, constraining photosynthate available for C sequestration in aboveground tree biomass. Second, greater N demand may increase Rh_‐decomp_ by increasing microbial oxidative enzyme production and enhancing Rh_‐rhizo_ by increasing plant rhizosphere C flux. Both of these effects could decrease soil C stocks. Overall, our results suggest that human activities that alter ecosystem N availability and demand will have substantial impacts on forest C cycling and thus global climate.

## Author Contributions

**David W. Frey:** conceptualization, data curation, formal analysis, funding acquisition, investigation, methodology, project administration, software, visualization, writing – original draft, writing – review and editing. **Eden Kebede:** investigation, methodology, writing – review and editing. **Jed P. Sparks:** methodology, resources, writing – review and editing. **Timothy J. Fahey:** methodology, writing – review and editing. **Christine L. Goodale:** conceptualization, data curation, funding acquisition, investigation, methodology, project administration, resources, supervision, writing – review and editing.

## Conflicts of Interest

The authors declare no conflicts of interest.

## Supporting information


Data S1.

Data S2.

## Data Availability

The data and R code that support the findings of this study are openly available in the Environmental Data Initiative data repository at https://doi.org/10.6073/pasta/573ee0dc6a98b30d5fa4a92dd44b8470, package identification number edi.1910.4.
