# Supplementary material for: Increased Soil Nitrogen Availability Suppresses Annual Soil Respiration in Mixed Temperate Forests Regardless of Acidification
Source: Glob Chang Biol. 2025 Apr 2;31(4):e70140. doi: 10.1111/gcb.70140 (PMC11963746; doi:10.1111/gcb.70140)
Supplement: Supplementary file 1 — Data S1. [file GCB-31-e70140-s002.pdf]

Increased soil nitrogen availability suppresses annual soil respiration in mixed temperate forests regardless of acidification

Authors: David W. Frey[1], Eden Kebede[1], Jed P. Sparks[1], Timothy J. Fahey[2], Christine L. Goodale[1]

Author Affiliations: [1]Department of Ecology and Evolutionary Biology, Cornell University, Ithaca, NY, USA; [2]Department of Natural Resources and the Environment, Cornell University, Ithaca, NY, USA

Correspondence: David W. Frey (dwf62@cornell.edu)

Supplemental Tables

Unrounded coefficients and statistics presented in Tables S1-S17 can be obtained using data and R code published in Frey et al. (2025).

Table S1: Type III F-tests for effects of treatments, stand age, their interaction, and sites on pre-treatment (2009-2010) 0-10 cm soil pH, 2019 0-10 cm soil pH (after 8 years of treatment) and 2019 resin available N (after 9 years of treatment).

**Pre-treatment Soil pH (2009-2010)**		
Predictor	*F*	*p*
Treatment	$F_{3, 12} = 0.55$	0.66
Stand Age	$F_{1, 2} = 3.0$	0.23
Site	$F_{2,2} = 3.0$	0.25
Treatment*Stand Age	$F_{3, 12} = 0.82$	0.51
**2019 Soil pH**		
Treatment	$F_{3, 14.1} = 11.8$	0.0004
Stand Age	$F_{1, 14.1} = 6.0$	0.03
Site	$F_{2, 14.1} = 5.0$	0.02
Treatment*Stand Age	$F_{3, 14.1} = 2.3$	0.12
**2019 Resin Available N (μg N g resin^{-1} day^{-1})**[†]		
Treatment	$F_{3, 11.9} = 7.6$	0.004
Stand Age	$F_{1, 2} = 0.54$	0.54
Site	$F_{2, 2} = 0.11$	0.90
Treatment*Stand Age	$F_{3, 11.9} = 1.2$	0.37

[†]Resin available N was natural log transformed for analysis

Table S2: Type III F-tests for effects of treatments, stand age, their interaction, sites, and soil water content on incubation C fluxes measured per g soil ($Rh_{-decomp/mass}$), per g soil C ($Rh_{-decomp/SOC}$), and per m^2 ($Rh_{-decomp/m2}$) from incubations with water added substituted for field moist soils in two stands where soils were particularly dry.

	**Flux per g Soil (μg CO_2-C g $soil^{-1}$ hr^{-1})**		**Flux per g Soil C (μg CO_2-C g SOC^{-1} hr^{-1})**		**Flux per m^2 (mg CO_2-C m^{-2} hr^{-1})[††]**	
			Forest Floor			
Predictor	*F*	*p*	*F*	*p*	*F*	*p*
Treatment (Trt)	$F_{3, 9.1} = 3.5$	0.06	$F_{3, 7.9} = 3.4$	0.08	$F_{3, 9} = 0.31$	0.82
Stand Age	$F_{1, 1} = 0.15$	0.76	$F_{1, 1} = 0.26$	0.70	$F_{1, 1} = 0.60$	0.58
Site	$F_{2, 2} = 0.07$	0.94	$F_{2, 2} = 0.03$	0.0006	$F_{2, 2} = 0.03$	0.97
SWC[†]	$F_{1, 79.6} = 36.6$	< 0.0001	$F_{1, 79.8} = 12.9$	0.97	$F_{1, 82.8} = 5.0$	0.03
Trt*Stand Age	$F_{3, 9} = 0.74$	0.56	$F_{3, 7.8} = 0.16$	0.92	$F_{3, 9} = 0.76$	0.55
			0-3 cm Mineral Soil			
Treatment (Trt)	$F_{3, 13.6} = 2.3$	0.12	$F_{3, 104} = 6.7$	0.0003	$F_{3, 12} = 2.6$	0.10
Stand Age	$F_{1, 13.6} = 28.6$	0.0001	$F_{1, 2} = 5.0$	0.15	$F_{1, 2} = 4.0$	0.18
Site	$F_{2, 16.4} = 7.7$	0.004	$F_{2, 2.1} = 0.38$	0.72	$F_{2, 2.1} = 0.06$	0.95
SWC[†]	$F_{1, 93.7} = 27.1$	< 0.0001	$F_{1, 104.6} = 3.0$	0.09	$F_{1, 84.8} = 0.003$	0.96
Trt*Stand Age	$F_{3, 13.4} = 1.9$	0.18	$F_{3, 104} = 1.3$	0.27	$F_{3, 11.7} = 0.92$	0.46
			3-10 cm Mineral Soil			
Treatment (Trt)	$F_{3, 12.2} = 2.7$	0.09	$F_{3, 12.4} = 7.4$	0.004	$F_{3, 12.2} = 1.6$	0.25
Stand Age	$F_{1, 2} = 3.0$	0.23	$F_{1, 2} = 3.6$	0.20	$F_{1, 2} = 2.5$	0.26
Site	$F_{2, 2} = 0.43$	0.70	$F_{2, 2} = 0.18$	0.84	$F_{2, 2} = 0.21$	0.82
SWC[†]	$F_{1, 103} = 4.1$	0.047	$F_{1, 103.3} = 0.36$	0.55	$F_{1, 102.5} = 0.13$	0.72
Trt*Stand Age	$F_{3, 12.1} = 1.1$	0.39	$F_{3, 12.2} = 2.3$	0.13	$F_{3, 12.1} = 0.27$	0.85

[†]SWC = Soil water content

[††]Forest floor and 3-10 cm $Rh_{-decomp/m2}$ were square root and natural log transformed for analysis, respectively

Table S3: Type III F-tests for effects of treatments, stand age, their interaction, sites, and soil water content on incubation C fluxes measured per g soil ($Rh_{-decomp/mass}$), per g soil C ($Rh_{-decomp/SOC}$), and per m^2 ($Rh_{-decomp/m2}$) from incubations using field moist soils from all stands.

	**Flux per g Soil (μg CO_2-C g $soil^{-1}$ hr^{-1})**		**Flux per g Soil C (μg CO_2-C g SOC^{-1} hr^{-1})**		**Flux per m^2 (mg CO_2-C m^{-2} hr^{-1})††**	
			Forest Floor			
Predictor	*F*	*p*	*F*	*p*	*F*	*p*
Treatment (Trt)	$F_{3, 9.4} = 3.0$	0.09	$F_{3, 9} = 3.1$	0.08	$F_{3, 9.2} = 0.34$	0.79
Stand Age	$F_{1, 1} = 0.18$	0.74	$F_{1, 1} = 0.31$	0.68	$F_{1, 1} = 0.66$	0.57
Site	$F_{2, 2} = 0.06$	0.94	$F_{2, 2} = 0.02$	0.98	$F_{2, 2} = 0.009$	0.99
SWC†	$F_{1, 77.5} = 43.3$	< 0.0001	$F_{1, 79.4} = 17.4$	< 0.0001	$F_{1, 81.9} = 6.0$	0.02
Trt*Stand Age	$F_{3, 9.3} = 1.1$	0.40	$F_{3, 8.9} = 0.97$	0.45	$F_{3, 9.1} = 1.25$	0.35
			0-3 cm Mineral Soil			
Treatment (Trt)	$F_{3, 13.2} = 4.3$	0.03	$F_{3, 11.9} = 6.1$	0.009	$F_{3, 12} = 1.6$	0.24
Stand Age	$F_{1, 13.2} = 53.1$	< 0.0001	$F_{1, 2} = 5.8$	0.14	$F_{1, 2} = 4.5$	0.17
Site	$F_{2, 16.4} = 5.9$	0.01	$F_{2, 2} = 0.31$	0.76	$F_{2, 2} = 0.01$	0.99
SWC†	$F_{1, 76.3} = 69.7$	< 0.0001	$F_{1, 77.7} = 18.4$	< 0.0001	$F_{1, 91.3} = 2.0$	0.16
Trt*Stand Age	$F_{3, 13} = 1.3$	0.32	$F_{3, 11.7} = 1.0$	0.42	$F_{3, 11.8} = 1.3$	0.31
			3-10 cm Mineral Soil			
Treatment (Trt)	$F_{3, 12.2} = 4.1$	0.03	$F_{3, 12.3} = 8.0$	0.003	$F_{3, 12.3} = 3.2$	0.06
Stand Age	$F_{1, 1.9} = 5.4$	0.15	$F_{1, 2} = 5.3$	0.15	$F_{1, 1.9} = 5.3$	0.15
Site	$F_{2, 2} = 0.36$	0.73	$F_{2, 2} = 0.25$	0.80	$F_{2, 2} = 0.02$	0.98
SWC†	$F_{1, 102.3} = 14.1$	0.0003	$F_{1, 105.4} = 1.3$	0.26	$F_{1, 105.4} = 6.1$	0.02
Trt*Stand Age	$F_{3, 12} = 0.86$	0.49	$F_{3, 12.1} = 1.4$	0.30	$F_{3, 12.1} = 0.19$	0.90

†SWC = Soil water content

††Forest floor and 3-10 cm $Rh_{-decomp/m2}$ were square root transformed for analysis

Table S4: Type III F-tests for effects of treatments, stand age, their interaction, site, soil temperature, months, and the interaction between months and treatments on soil respiration (Rsoil).

**Monthly Soil Respiration (mg C m^{-2} hr^{-1})**		
Predictor	*F*	*p*
Treatment	$F_{3, 12}$ = 4.6	0.02
Stand Age	$F_{1, 2}$ = 1.9	0.30
Month	$F_{5, 538}$ = 53.1	< 0.0001
Soil Temperature	$F_{1, 538}$ = 34.3	< 0.0001
Site	$F_{2,2}$ = 0.55	0.65
Treatment*Stand Age	$F_{3, 12}$ = 0.89	0.47
Treatment*Month	$F_{15, 538}$ = 2.1	0.007
**Annual Soil Respiration (Mg C ha^{-1} yr^{-1})**		
Treatment	$F_{3, 12}$ = 3.9	0.04
Stand Age	$F_{1, 2}$ = 0.82	0.46
Site	$F_{2, 2}$ = 0.87	0.53
Treatment*Stand Age	$F_{3, 12}$ = 0.83	0.50

Table S5: Estimated marginal means (± SEs) for pre-treatment 0-10 cm mineral soil pH (2009-2010), soil pH in the same layer in 2019 (after 8 years of treatment), and 2019 resin available N (after 9 years of treatment) obtained from linear mixed effects models and contrasts between treatments. Results for overall acidification and +N effects are averaged across both treatments (($(NH_4)_2SO_4$, S; and $NaNO_3$, $(NH_4)_2SO_4$, respectively) and only displayed when effects of individual N or acidification treatments are directionally consistent relative to the control.

**Pre-treatment Soil pH (2009-2010)**				
Treatment	*Emmean*	*Contrast*	*Difference*	*p*
Control	4.33 ± 0.14	$NaNO_3$ - Control	-0.24	0.63
$NaNO_3$	4.09 ± 0.14	$(NH_4)_2$SO4 - Control	-0.20	0.76
$(NH_4)_2SO_4$	4.14 ± 0.14	Sulfur - Control	-0.14	0.89
S	4.19 ± 0.14	$(NH_4)_2SO_4$ - $NaNO_3$	0.05	1.00
-	-	S - $NaNO_3$	0.10	0.96
-	-	S- $(NH_4)_2SO_4$	0.06	0.99
**2019 Soil pH**				
Control	4.67 ± 0.10	$NaNO_3$ - Control	0.30	0.22
$NaNO_3$	4.98 ± 0.11	$(NH_4)_2SO_4$ - Control	-0.41	0.07
$(NH_4)_2SO_4$	4.26 ± 0.11	Sulfur - Control	-0.45	0.04
S	4.22 ± 0.11	$(NH_4)_2SO_4$ - $NaNO_3$	-0.72	0.002
-	-	S - $NaNO_3$	-0.76	0.001
-	-	S- $(NH_4)_2SO_4$	-0.04	0.99
+N	-	+N - Control	-	-
Acidification	4.24 ± 0.07	Acidification - Control	-0.43	0.006
**2019 Resin Available N (µg N g resin^{-1} day^{-1})†**				
	Geometric Mean	*Contrast*	*Ratio*	*p*
Control	1.90 ± 1.26	$NaNO_3$ / Control	2.37	0.24
$NaNO_3$	4.50 ± 2.98	$(NH_4)_2SO_4$ / Control	2.94	0.11
$(NH_4)_2SO_4$	5.59 ± 3.70	Sulfur / Control	0.49	0.37
S	0.92 ± 0.61	$(NH_4)_2SO_4$ / $NaNO_3$	1.24	0.96
-	-	S / $NaNO_3$	0.20	0.01
-	-	S / $(NH_4)_2SO_4$	0.17	0.006
+N	5.05 ± 3.17	+N / Control	2.64	0.02
Acidification	-	Acidification - Control	-	-

†Resin available N was natural log transformed for analysis

Table S6: Estimated marginal means (± SEs) and contrasts between treatments for incubation C flux per g soil ($Rh_{-decomp/mass}$) in the forest floor, 0-3 cm mineral soils, and 3-10 cm mineral soils from incubations with water added substituted for field moist soils in two stands where soils were particularly dry. Estimates are obtained from linear mixed effects models. Results for overall acidification and +N effects are averaged across both treatments ($(NH_4)_2SO_4$, S; and $NaNO_3$, $(NH_4)_2SO_4$, respectively) and only displayed when effects of individual N or acidification treatments are directionally consistent relative to the control.

**Forest Floor Flux (µg CO_2-C g soil^{-1} hr^{-1})**				
Treatment	*Emmean*	*Contrast*	*Difference*	*p*
Control	8.84 ± 2.39	$NaNO_3$ - Control	-2.57	0.17
$NaNO_3$	6.27 ± 2.39	$(NH_4)_2SO_4$ - Control	-3.37	0.06
$(NH_4)_2SO_4$	5.47 ± 2.38	Sulfur - Control	-2.55	0.18
S	6.29 ± 2.38	$(NH_4)_2SO_4$ - $NaNO_3$	-0.80	0.89
-	-	S - $NaNO_3$	0.01	1.00
-	-	S - $(NH_4)_2SO_4$	0.82	0.88
+N	5.87 ± 2.32	+N - Control	-2.97	0.02
Acidification	5.88 ± 2.32	Acidification - Control	-2.96	0.02
**0-3 cm Mineral Soil Flux (µg CO_2-C g soil^{-1} hr^{-1})**				
Control	1.03 ± 0.07	$NaNO_3$ - Control	-0.04	0.98
$NaNO_3$	0.99 ± 0.07	$(NH_4)_2SO_4$ - Control	-0.21	0.18
$(NH_4)_2SO_4$	0.81 ± 0.07	Sulfur - Control	0.006	0.99
S	1.03 ± 0.07	$(NH_4)_2SO_4$ - $NaNO_3$	-0.18	0.31
-	-	S - $NaNO_3$	0.04	0.97
-	-	S - $(NH_4)_2SO_4$	0.22	0.15
+N	0.90 ± 0.05	+N - Control	-0.13	0.16
Acidification	-	Acidification - Control	-	-
**3-10 cm Mineral Soil Flux (µg CO_2-C g soil^{-1} hr^{-1})**				
Control	0.39 ± 0.06	$NaNO_3$ - Control	-0.009	0.99
$NaNO_3$	0.39 ± 0.06	$(NH_4)_2SO_4$ - Control	-0.06	0.74
$(NH_4)_2SO_4$	0.33 ± 0.06	Sulfur - Control	0.11	0.35
S	0.50 ± 0.06	$(NH_4)_2SO_4$ - $NaNO_3$	-0.06	0.81
-	-	S - $NaNO_3$	0.12	0.29
-	-	S - $(NH_4)_2SO_4$	0.17	0.07
+N	0.36 ± 0.05	+N - Control	-0.04	0.51
Acidification	-	Acidification - Control	-	-

Table S7: Estimated marginal means (± SEs) and contrasts between treatments for incubation C flux per g soil C ($Rh_{-decomp/SOC}$) in the forest floor, 0-3 cm mineral soils, and 3-10 cm mineral soils from incubations with water added substituted for field moist soils in two stands where soils were particularly dry. Estimates are obtained from linear mixed effects models. Results for overall acidification and +N effects are averaged across both treatments (($(NH_4)_2SO_4$, S; and $NaNO_3$, $(NH_4)_2SO_4$, respectively) and only displayed when effects of individual N or acidification treatments are directionally consistent relative to the control.

**Forest Floor Flux (µg CO_2-C g SOC^{-1} hr^{-1})**				
Treatment	*Emmean*	*Contrast*	*Difference*	*p*
Control	25.7 ± 6.56	$NaNO_3$ - Control	-7.86	0.13
$NaNO_3$	17.9 ± 6.54	$(NH_4)_2SO_4$ - Control	-8.55	0.09
$(NH_4)_2SO_4$	17.2 ± 6.53	Sulfur - Control	-8.14	0.11
S	17.6 ± 6.52	$(NH_4)_2SO_4$ - $NaNO_3$	-0.69	1.00
-	-	S - $NaNO_3$	-0.28	1.00
-	-	S - $(NH_4)_2SO_4$	0.40	1.00
+N	17.5 ± 6.34	+N - Control	-8.20	0.02
Acidification	17.4 ± 6.33	Acidification - Control	-8.34	0.02
**0-3 cm Mineral Soil Flux (µg CO_2-C g SOC^{-1} hr^{-1})**				
Control	15.9 ± 1.94	$NaNO_3$ - Control	-1.05	0.83
$NaNO_3$	14.8 ± 1.95	$(NH_4)_2SO_4$ - Control	-5.41	0.005
$(NH_4)_2SO_4$	10.4 ± 1.96	Sulfur - Control	-2.00	0.42
S	13.8 ± 1.94	$(NH_4)_2SO_4$ - $NaNO_3$	-4.36	0.02
-	-	S - $NaNO_3$	-0.95	0.87
-	-	S - $(NH_4)_2SO_4$	3.41	0.08
+N	12.6 ± 1.85	+N - Control	-3.23	0.02
Acidification	12.1 ± 1.85	Acidification - Control	-3.71	0.009
**3-10 cm Mineral Soil Flux (µg CO_2-C g SOC^{-1} hr^{-1})**				
Control	12.2 ± 2.11	$NaNO_3$ - Control	-1.24	0.73
$NaNO_3$	10.9 ± 2.10	$(NH_4)_2SO_4$ - Control	-3.23	0.08
$(NH_4)_2SO_4$	8.95 ± 2.11	Sulfur - Control	2.25	0.29
S	14.4 ± 2.11	$(NH_4)_2SO_4$ - $NaNO_3$	-1.99	0.38
-		S - $NaNO_3$	3.48	0.053
-		S - $(NH_4)_2SO_4$	5.47	0.003
+N	9.95 ± 2.02	+N - Control	-2.23	0.051
Acidification	-	Acidification - Control	-	-

Table S8: Coefficients, type III F-tests, and marginal R^2 from linear mixed effects models examining effects of resin available N (natural log-transformed), soil pH, and sites on incubation C fluxes ($Rh_{-decomp/SOC}$; µg CO_2-C g SOC^{-1} hr^{-1}) in the forest floor, 0-3 cm mineral soils, and 3-10 cm mineral soils.

Forest Floor

Predictor	*Coefficient*	*SE*	*p*
Intercept	-22.22	7.81	0.02
Resin Available N	-3.07	0.66	0.0004
2019 Soil pH	12.23	1.58	< 0.0001
Site - Carter Creek	-3.60	6.86	0.65
Site - Mt. Pleasant	-11.38	8.44	0.31

Predictor	*F*	*p*	*Marginal R2*
Resin Available N	$F_{1, 14.2} = 21.8$	0.0004	0.22
2019 Soil pH	$F_{1, 13.1} = 60.0$	< 0.0001	0.20
Site	$F_{2, 2} = 0.91$	0.52	0.007

0-3 cm Mineral Soil

Predictor	*Coefficient*	*SE*	*p*
Intercept	-9.73	7.46	0.21
Resin Available N	-1.42	0.60	0.03
2019 Soil pH	6.32	1.80	0.003
Site - Carter Creek	0.08	4.29	0.99
Site - Mt. Pleasant	-2.92	4.40	0.55

Predictor	*F*	*p*	*Marginal R2*
Resin Available N	$F_{1, 18.9} = 5.7$	0.03	0.13
2019 Soil pH	$F_{1, 17.3} = 12.4$	0.003	0.19
Site	$F_{2, 2.8} = 0.31$	0.76	-0.03

3-10 cm Mineral Soil

Predictor	*Coefficient*	*SE*	*p*
Intercept	10.79	8.46	0.22
Resin Available N	-2.23	0.60	0.001
2019 Soil pH	0.98	1.88	0.61
Site - Carter Creek	1.14	4.43	0.82
Site - Mt. Pleasant	-2.59	4.48	0.61

Predictor	*F*	*p*	*Marginal R2*
Resin Available N	$F_{1, 18.7} = 13.8$	0.001	0.29
2019 Soil pH	$F_{1, 17.3} = 0.27$	0.61	0.01
Site	$F_{2, 2.7} = 0.37$	0.72	0.002

Table S9: Estimated marginal means (± SEs) and contrasts between treatments for incubation C flux per m^2 ($Rh_{-decomp/m2}$) in the forest floor, 0-3 cm mineral soils, and 3-10 cm mineral soils from incubations with water added substituted for field moist soils in two stands where soils were particularly dry. Estimates are obtained from linear mixed effects models. Results for overall acidification and +N effects are averaged across both treatments (($(NH_4)_2SO_4$, S; and $NaNO_3$, $(NH_4)_2SO_4$, respectively) and only displayed when effects of individual N or acidification treatments are directionally consistent relative to the control.

**Forest Floor Flux (mg CO_2-C m^{-2} hr^{-1})†**				
Treatment	*Emmean*	*Contrast*	*Difference*	*p*
Control	22.3 ± 9.02	$NaNO_3$ - Control	-0.76	0.80
$NaNO_3$	15.7 ± 7.59	$(NH_4)_2SO_4$ - Control	-0.60	0.89
$(NH_4)_2SO_4$	17.0 ± 7.89	Sulfur - Control	-0.43	0.95
S	18.4 ± 8.20	$(NH_4)_2SO_4$ - $NaNO_3$	0.16	1.00
-	-	S - $NaNO_3$	0.33	0.98
-	-	S - $(NH_4)_2SO_4$	0.17	1.00
+N	16.3 ± 6.96	+N - Control	-0.68	0.49
Acidification	17.7 ± 7.24	Acidification - Control	-0.52	0.63
**0-3 cm Mineral Soil Flux (mg CO_2-C m^{-2} hr^{-1})**				
Control	17.4 ± 2.04	$NaNO_3$ - Control	-0.78	0.97
$NaNO_3$	16.6 ± 2.04	$(NH_4)_2SO_4$ - Control	-4.77	0.09
$(NH_4)_2SO_4$	12.7 ± 2.06	Sulfur - Control	-1.78	0.76
S	15.6 ± 2.04	$(NH_4)_2SO_4$ - $NaNO_3$	-3.99	0.18
-	-	S - $NaNO_3$	-1.00	0.94
-	-	S - $(NH_4)_2SO_4$	2.99	0.39
+N	14.6 ± 1.84	+N - Control	-2.77	0.15
Acidification	14.1 ± 1.84	Acidification - Control	-3.27	0.09
**3-10 cm Mineral Soil Flux (mg CO_2-C m^{-2} hr^{-1})††**				
	Geometric Mean	*Contrast*	*Ratio*	*p*
Control	15.6 ± 2.93	$NaNO_3$ / Control	0.86	0.81
$NaNO_3$	13.3 ± 2.50	$(NH_4)_2SO_4$ / Control	0.79	0.55
$(NH_4)_2SO_4$	12.3 ± 2.32	Sulfur / Control	1.11	0.93
S	17.3 ± 3.25	$(NH_4)_2SO_4$ / $NaNO_3$	0.92	0.97
-	-	S / $NaNO_3$	1.30	0.47
-	-	S / $(NH_4)_2SO_4$	1.40	0.26
+N	12.8 ± 2.14	+N / Control	0.82	0.22
Acidification	-	Acidification / Control	-	-

†Forest floor fluxes were square root transformed for analysis. Estimates and standard errors are back-transformed to the original scale, but differences are reported on the square root-transformed scale.
††3-10 cm mineral soil fluxes were natural log transformed for analysis.

Table S10: Estimated marginal means (± SEs) and contrasts between treatments for incubation C flux per g soil ($Rh_{-decomp/mass}$) in the forest floor, 0-3 cm mineral soils, and 3-10 cm mineral soils from incubations using field moist soils from all stands. Estimates are obtained from linear mixed effects models. Results for overall acidification and +N effects are averaged across both treatments (($(NH_4)_2SO_4$, S; and $NaNO_3$, $(NH_4)_2SO_4$, respectively) and only displayed when effects of individual N or acidification treatments are directionally consistent relative to the control.

**Forest Floor Flux (μg CO_2-C g soil^{-1} hr^{-1})**				
Treatment	*Emmean*	*Contrast*	*Difference*	*p*
Control	8.22 ± 2.58	$NaNO_3$ - Control	-2.46	0.19
$NaNO_3$	5.75 ± 2.57	$(NH_4)_2SO_4$ - Control	-3.09	0.08
$(NH_4)_2SO_4$	5.12 ± 2.57	Sulfur - Control	-2.16	0.27
S	6.06 ± 2.57	$(NH_4)_2SO_4$ - $NaNO_3$	-0.63	0.94
-	-	S - $NaNO_3$	0.31	0.99
-	-	S - $(NH_4)_2SO_4$	0.94	0.83
+N	5.44 ± 2.51	+N - Control	-2.78	0.03
Acidification	5.59 ± 2.51	Acidification - Control	-2.63	0.03
**0 - 3 cm Mineral Soil Flux (μg CO_2-C g soil^{-1} hr^{-1})**				
Control	0.93 ± 0.06	$NaNO_3$ - Control	-0.07	0.83
$NaNO_3$	0.85 ± 0.06	$(NH_4)_2SO_4$ - Control	-0.18	0.20
$(NH_4)_2SO_4$	0.75 ± 0.06	Sulfur - Control	0.11	0.60
S	1.03 ± 0.06	$(NH_4)_2SO_4$ - $NaNO_3$	-0.11	0.58
-	-	S - $NaNO_3$	0.18	0.19
-	-	S - $(NH_4)_2SO_4$	0.28	0.02
+N	0.80 ± 0.04	+N - Control	-0.13	0.11
Acidification	-	Acidification - Control	-	-
**3 - 10 cm Mineral Soil Flux (μg CO_2-C g soil^{-1} hr^{-1})**				
Control	0.34 ± 0.06	$NaNO_3$ - Control	-0.03	0.96
$NaNO_3$	0.31 ± 0.06	$(NH_4)_2SO_4$ - Control	-0.04	0.87
$(NH_4)_2SO_4$	0.30 ± 0.06	Sulfur - Control	0.14	0.14
S	0.48 ± 0.06	$(NH_4)_2SO_4$ - $NaNO_3$	-0.01	0.99
-	-	S - $NaNO_3$	0.16	0.06
-	-	S - $(NH_4)_2SO_4$	0.18	0.04
+N	0.31 ± 0.05	+N - Control	-0.04	0.48
Acidification	-	Acidification - Control	-	-

Table S11: Estimated marginal means (± SEs) and contrasts between treatments for incubation C flux per g soil C ($Rh_{-decomp/SOC}$) in the forest floor, 0-3 cm mineral soils, and 3-10 cm mineral soils from incubations using field moist soils from all stands. Estimates are obtained from linear mixed effects models. Results for overall acidification and +N effects are averaged across both treatments (($(NH_4)_2SO_4$, S; and $NaNO_3$, $(NH_4)_2SO_4$, respectively) and only displayed when effects of individual N or acidification treatments are directionally consistent relative to the control.

**Forest Floor Flux (µg CO_2-C g SOC^{-1} hr^{-1})**				
Treatment	*Emmean*	*Contrast*	*Difference*	*p*
Control	22.1 ± 7.19	$NaNO_3$ - Control	-6.54	0.13
$NaNO_3$	15.6 ± 7.17	$(NH_4)_2SO_4$ - Control	-6.83	0.10
$(NH_4)_2SO_4$	15.3 ± 7.17	Sulfur - Control	-5.90	0.18
S	16.2 ± 7.16	$(NH_4)_2SO_4$ - $NaNO_3$	-0.29	1.00
-	-	S - $NaNO_3$	0.64	0.99
-	-	S - $(NH_4)_2SO_4$	0.93	0.98
+N	15.4 ± 7.05	+N - Control	-6.69	0.03
Acidification	15.7 ± 7.04	Acidification - Control	-6.37	0.03
**0 - 3 cm Mineral Soil (µg CO_2-C g SOC^{-1} hr^{-1})**				
Control	13.6 ± 2.22	$NaNO_3$ - Control	-1.24	0.68
$NaNO_3$	12.4 ± 2.22	$(NH_4)_2SO_4$ - Control	-3.99	0.02
$(NH_4)_2SO_4$	9.64 ± 2.22	Sulfur - Control	0.13	0.99
S	13.8 ± 2.21	$(NH_4)_2SO_4$ - $NaNO_3$	-2.75	0.11
-	-	S - $NaNO_3$	1.38	0.60
-	-	S - $(NH_4)_2SO_4$	4.12	0.01
+N	11.0 ± 2.15	+N - Control	-2.61	0.02
Acidification	-	Acidification - Control	-	-
**3 - 10 cm Mineral Soil (µg CO_2-C g SOC^{-1} hr^{-1})**				
Control	10.6 ± 2.35	$NaNO_3$ - Control	-1.59	0.56
$NaNO_3$	9.01 ± 2.34	$(NH_4)_2SO_4$ - Control	-2.56	0.21
$(NH_4)_2SO_4$	8.04 ± 2.35	Sulfur - Control	2.97	0.12
S	13.6 ± 2.34	$(NH_4)_2SO_4$ - $NaNO_3$	-0.97	0.85
-	-	S - $NaNO_3$	4.56	0.01
-	-	S - $(NH_4)_2SO_4$	5.53	0.003
+N	8.53 ± 2.27	+N - Control	-2.08	0.07
Acidification	-	Acidification - Control	-	-

Table S12: Estimated marginal means (± SEs) and contrasts between treatments for incubation C flux per m^2 ($Rh_{-decomp/m2}$) in the forest floor, 0-3 cm mineral soils, and 3-10 cm mineral soils from incubations using field moist soils from all stands. Estimates are obtained from linear mixed effects models. Results for overall acidification and +N effects are averaged across both treatments (($(NH_4)_2SO_4$, S; and $NaNO_3$, $(NH_4)_2SO_4$, respectively) and only displayed when effects of individual N or acidification treatments are directionally consistent relative to the control.

Treatment	Emmean	Contrast	Difference	p
**Forest Floor Flux (mg CO_2-C m^{-2} hr^{-1})†**				
Control	18.5 ± 9.34	$NaNO_3$ - Control	-0.67	0.79
$NaNO_3$	13.2 ± 7.89	$(NH_4)_2SO_4$ - Control	-0.49	0.90
$(NH_4)_2SO_4$	14.5 ± 8.30	Sulfur - Control	-0.20	0.99
S	16.8 ± 8.90	$(NH_4)_2SO_4$ - $NaNO_3$	0.18	0.99
-	-	S - $NaNO_3$	0.47	0.91
-	-	S - $(NH_4)_2SO_4$	0.29	0.98
+N	13.9 ± 7.63	+N - Control	-0.58	0.50
Acidification	15.7 ± 8.11	Acidification - Control	-0.34	0.75
**0 - 3 cm Mineral Soil Flux (mg CO_2-C m^{-2} hr^{-1})**				
Control	15.0 ± 2.59	$NaNO_3$ - Control	-1.03	0.95
$NaNO_3$	14.0 ± 2.58	$(NH_4)_2SO_4$ - Control	-3.36	0.35
$(NH_4)_2SO_4$	11.7 ± 2.58	Sulfur - Control	0.48	0.99
S	15.5 ± 2.57	$(NH_4)_2SO_4$ - $NaNO_3$	-2.32	0.63
-	-	S - $NaNO_3$	1.51	0.86
-	-	S - $(NH_4)_2SO_4$	3.84	0.24
+N	12.8 ± 2.40	+N - Control	-2.20	0.22
Acidification	-	Acidification - Control	-	-
**3 - 10 cm Mineral Soil Flux (mg CO_2-C m^{-2} hr^{-1})†**				
Control	12.5 ± 3.12	$NaNO_3$ - Control	-0.44	0.57
$NaNO_3$	9.62 ± 2.73	$(NH_4)_2SO_4$ - Control	-0.20	0.93
$(NH_4)_2SO_4$	11.1 ± 2.94	Sulfur - Control	0.56	0.39
S	16.8 ± 3.60	$(NH_4)_2SO_4$ - $NaNO_3$	0.23	0.90
-	-	S - $NaNO_3$	0.99	0.051
-	-	S - $(NH_4)_2SO_4$	0.76	0.16
+N	10.4 ± 2.62	+N - Control	-0.32	0.29
Acidification	-	Acidification - Control	-	-

†Forest floor and 3-10 cm mineral soil fluxes were square root transformed for analysis. Estimates and standard errors are back-transformed to the original scale, but differences are reported on the square root-transformed scale.

Table S13: Estimated marginal means (± SEs) for soil respiration (mg C m^{-2} hr^{-1}) obtained from linear mixed effects models and contrasts between treatments by month.

		**October 2020**		
Treatment	*Emmean*	*Contrast*	*Difference*	*p*
Control	161 ± 17.0	$NaNO_3$ - Control	-5.83	0.99
$NaNO_3$	155 ± 17.0	$(NH_4)_2SO4$ - Control	-18.3	0.70
$(NH_4)_2SO_4$	142 ± 16.9	Sulfur - Control	-17.3	0.74
S	143 ± 17.0	$(NH_4)_2SO_4$ - $NaNO_3$	-12.4	0.88
-	-	S - $NaNO_3$	-11.5	0.90
-	-	S - $(NH_4)_2SO_4$	0.95	1.00
		**April 2021**		
Control	144 ± 21.1	$NaNO_3$ - Control	-3.82	1.00
$NaNO_3$	140 ± 21.0	$(NH_4)_2SO4$ - Control	-12.5	0.88
$(NH_4)_2SO_4$	132 ± 20.7	Sulfur - Control	-12.9	0.87
S	131 ± 20.8	$(NH_4)_2SO_4$ - $NaNO_3$	-8.72	0.95
-	-	S - $NaNO_3$	-9.13	0.95
-	-	S - $(NH_4)_2SO_4$	-0.41	1.00
		**June 2021**		
Control	171 ± 16.7	$NaNO_3$ - Control	-21.2	0.62
$NaNO_3$	150 ± 16.7	$(NH_4)_2SO4$ - Control	-23.3	0.55
$(NH_4)_2SO_4$	148 ± 16.8	Sulfur - Control	-6.11	0.98
S	165 ± 16.8	$(NH_4)_2SO_4$ - $NaNO_3$	-2.20	1.00
-	-	S - $NaNO_3$	15.0	0.82
-	-	S - $(NH_4)_2SO_4$	17.2	0.75
		**July 2021**		
Control	292 ± 19.6	$NaNO_3$ - Control	-75.8	0.01
$NaNO_3$	216 ± 19.7	$(NH_4)_2SO4$ - Control	-82.7	0.007
$(NH_4)_2SO_4$	209 ± 19.8	Sulfur - Control	-7.39	0.98
S	285 ± 19.8	$(NH_4)_2SO_4$ - $NaNO_3$	-6.90	0.99
-	-	S - $NaNO_3$	68.4	0.02
-	-	S - $(NH_4)_2SO_4$	75.3	0.01

Table S13 (continued)

**August 2021**				
Control	214 ± 18.7	$NaNO_3$ - Control	-52.0	0.048
$NaNO_3$	162 ± 18.6	$(NH_4)_2$SO4 - Control	-64.8	0.01
$(NH_4)_2SO_4$	150 ± 18.9	Sulfur - Control	-18.3	0.72
S	196 ± 18.8	$(NH_4)_2SO_4$ - $NaNO_3$	-12.8	0.88
-	-	S - $NaNO_3$	33.7	0.26
-	-	S - $(NH_4)_2SO_4$	46.5	0.08
**October 2021**				
Control	148 ± 16.4	$NaNO_3$ - Control	-37.6	0.16
$NaNO_3$	110 ± 16.4	$(NH_4)_2$SO4 - Control	-46.7	0.06
$(NH_4)_2SO_4$	101 ± 16.4	Sulfur - Control	-0.73	1.00
S	147 ± 16.4	$(NH_4)_2SO_4$ - $NaNO_3$	-9.13	0.94
-	-	S - $NaNO_3$	36.9	0.17
-	-	S - $(NH_4)_2SO_4$	46.0	0.07

Table S14: Estimated marginal means (± SEs) for annual soil respiration (Mg C ha^{-1} yr^{-1}) obtained from linear mixed effects models and contrasts between treatments for models with and without $Rh_{-decomp/m2}$ included as a predictor. Results for overall acidification and +N effects are averaged across both treatments (($(NH_4)_2SO_4$, S; and $NaNO_3$, $(NH_4)_2SO_4$, respectively) and only displayed when effects of individual N or acidification treatments are directionally consistent relative to the control.

Annual Soil Respiration (Mg C ha^{-1} yr^{-1})

Treatment	*Emmean*	*Contrast*	*Difference*	*p*
Control	11.22 ± 0.85	$NaNO_3$ - Control	-1.51	0.19
$NaNO_3$	9.71 ± 0.85	$(NH_4)_2$SO4 - Control	-2.09	0.048
$(NH_4)_2SO_4$	9.13 ± 0.85	Sulfur - Control	-0.36	0.96
S	10.87 ± 0.85	$(NH_4)_2SO_4$ - $NaNO_3$	-0.58	0.84
-	-	S - $NaNO_3$	1.16	0.39
-	-	S- $(NH_4)_2SO_4$	1.74	0.11
+N	9.42 ± 0.78	+N - Control	-1.80	0.02
Acidification	10.00 ± 0.78	Acidification - Control	-1.22	0.097

Annual Soil Respiration (Mg C ha^{-1} yr^{-1}) with $Rh_{-decomp}$

Treatment	*Emmean*	*Contrast*	*Difference*	*p*
Control	10.86 ± 0.48	$NaNO_3$ - Control	-1.07	0.16
$NaNO_3$	9.79 ± 0.47	$(NH_4)_2SO_4$ - Control	-1.17	0.16
$(NH_4)_2SO_4$	9.70 ± 0.49	Sulfur - Control	-0.27	0.94
S	10.59 ± 0.47	$(NH_4)_2SO_4$ - $NaNO_3$	-0.09	1.00
-	-	S - $NaNO_3$	0.81	0.37
-	-	S- $(NH_4)_2SO_4$	0.90	0.34
+N	9.74 ± 0.41	+N - Control	-1.12	0.03
Acidification	10.14 ± 0.41	Acidification - Control	-0.72	0.16

Table S15: Coefficients, type III F-tests, and marginal R^2 from linear mixed effects models examining effects of resin available N (natural log-transformed), 2019 soil pH, and sites on annual soil respiration (Mg C ha^{-1} yr^{-1}).

Predictor	*Coefficient*	*SE*	*p*
Intercept	8.21	2.13	0.001
Resin Available N	-1.19	0.16	< 0.0001
2019 Soil pH	0.64	0.48	0.20
Site - Carter Creek	1.19	0.50	0.03
Site - Mt. Pleasant	0.84	0.54	0.13
Predictor	*F*	*p*	*Marginal R^2*
Resin Available N	$F_{1, 19}$ = 58.7	< 0.0001	0.66
2019 Soil pH	$F_{1, 19}$ = 1.8	0.20	0.01
Site	$F_{2, 19}$ = 2.8	0.08	0.11

Table S16: Coefficients, type III F-tests, and marginal R^2 from a linear mixed effects model examining effects of $Rh_{-decomp/m2}$ and sites on annual soil respiration (Mg C ha^{-1} yr^{-1}).

Predictor	*Coefficient*	*SE*	*p*
Intercept	4.01	1.11	0.01
$Rh_{-decomp/m2}$	0.09	0.02	< 0.0001
Site - Carter Creek	1.81	1.06	0.22
Site - Mt. Pleasant	2.91	1.07	0.097
Predictor	*F*	*p*	*Marginal R^2*
$Rh_{-decomp/m2}$	$F_{1, 13.3}$ = 35.2	< 0.0001	0.54
Site	$F_{2, 2.3}$ = 3.8	0.19	0.17

Table S17: Coefficients, type III F-tests, and marginal R^2 from a linear mixed effects model examining effects of treatments, $Rh_{-decomp/m2}$, and sites on annual soil respiration (Mg C ha^{-1} yr^{-1}).

Predictor	*Coefficient*	*SE*	*p*
Intercept	5.47	1.11	0.002
$NaNO_3$	-1.07	0.48	0.04
$(NH_4)_2SO_4$	-1.17	0.51	0.04
S	-0.27	0.47	0.58
$Rh_{-decomp/m2}$	0.08	0.02	0.0004
Site - Carter Creek	1.85	0.90	0.14
Site - Mt. Pleasant	2.78	0.91	0.06
Predictor	*F*	*p*	*Marginal R^2*
$Rh_{-decomp/m2}$	$F_{1, 10.3} = 26.2$	0.0004	0.48
Treatment	$F_{3, 15.1} = 2.6$	0.09	0.08
Site	$F_{2, 2.7} = 4.8$	0.13	0.18

Supplemental Figures

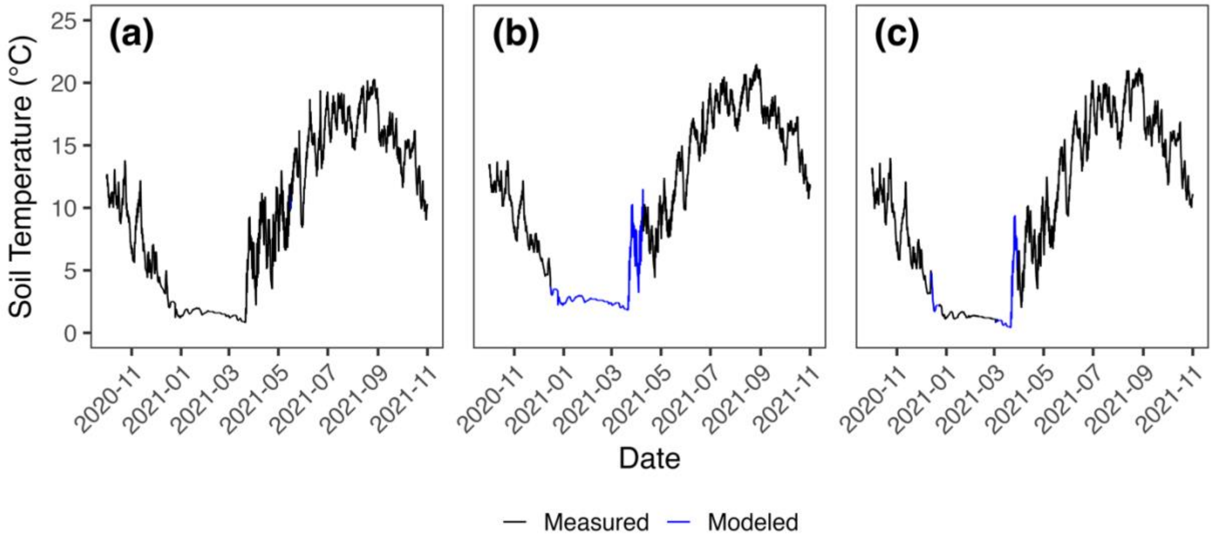

Figure S1: Soil temperature measured at 10 cm depth at Bald Hill (a), Carter Creek (b), and Mount Pleasant (c) sites from October 2020 to November 2021.

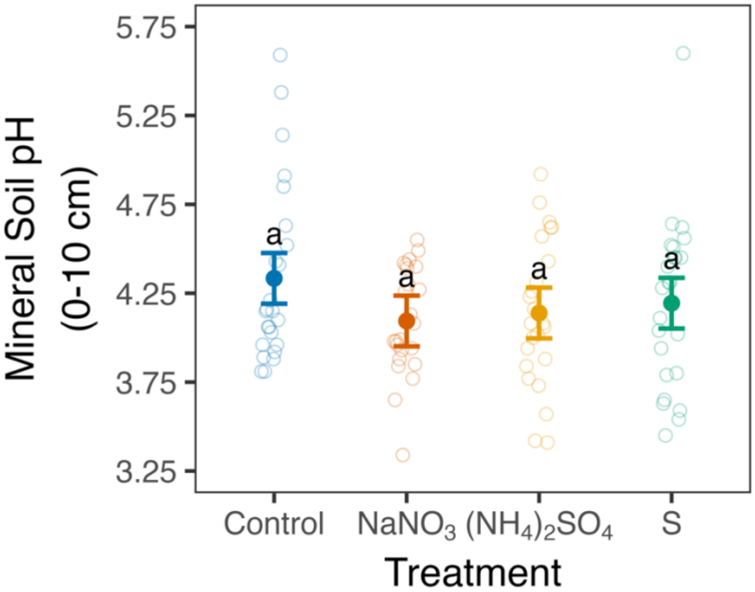

Figure S2: Pre-treatment (2009-2010) surface mineral soil (0-10 cm) pH by treatment. Solid points are estimated marginal means and error bars are associated standard errors estimated from a linear mixed effects model. Hollow points are measured values. Differences between treatments are statistically significant ($p \leq 0.05$) when treatments do not share common letters. Treatments are designated on the x-axis as: 1) control = no fertilization; 2) $NaNO_3$ = sodium nitrate addition; 3) $(NH_4)_2SO_4$ = ammonium sulfate addition; and 4) S = elemental sulfur addition. Note that pre-treatment pH was measured on dried soils while soil pH shown in Fig.1 (after 8 years of treatment) was measured on field moist soils, so direct comparisons of these values should not be made.

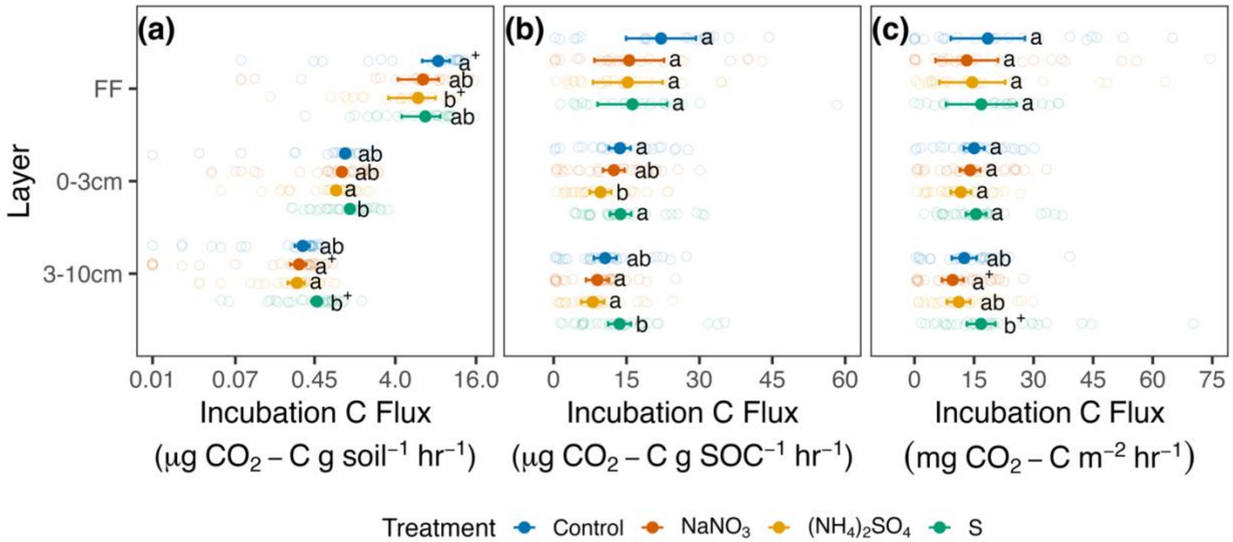

Figure S3: Forest floor (FF), 0-3 cm mineral soil, and 3-10 cm mineral soil incubation C fluxes per gram soil (a; $Rh_{-decomp/mass}$), per g soil organic carbon (SOC) (b; $Rh_{-decomp/SOC}$), and per m^2 (c; $Rh_{-decomp/area}$), using field moist soils from all stands. Solid points are estimated marginal means or back-transformed estimated marginal means when response variables were transformed to meet model assumptions. Error bars are associated standard errors. Hollow points are measured fluxes. Compact letters designate statistically significant ($p \leq 0.05$) differences between treatments within each depth separately, not differences between depths. Differences between treatments are statistically significant ($p \leq 0.05$) when treatments do not share common letters. When treatments do not share common letters and share + symbols, $0.1 \geq p > 0.05$.

References

Frey, D. W., Kebede, E. Sparks, J. P., Fahey, T. J., & Goodale, C. L. (2025). Soil nitrogen availability vs. acidification: effects on soil respiration, heterotrophic respiration, and soil physicochemical properties in mixed temperate forests in central New York, USA (2019-2022) ver 4. *Environmental Data Initiative.* https://doi.org/10.6073/pasta/573ee0dc6a98b30d5fa4a92dd44b8470.
