# Supplementary material for: Increased Soil Nitrogen Availability Suppresses Annual Soil Respiration in Mixed Temperate Forests Regardless of Acidification
Source: Glob Chang Biol. 2025 Apr 2;31(4):e70140. doi: 10.1111/gcb.70140 (PMC11963746; doi:10.1111/gcb.70140)
Supplement: Supplementary file 2 — Data S2. [file GCB-31-e70140-s001.pdf]

Increased soil nitrogen availability suppresses annual soil respiration in mixed temperate forests regardless of acidification

Authors: David W. Frey[1], Eden Kebede[1], Jed P. Sparks[1], Timothy J. Fahey[2], Christine L. Goodale[1]

Author Affiliations: [1]Department of Ecology and Evolutionary Biology, Cornell University, Ithaca, NY, USA; [2]Department of Natural Resources and the Environment, Cornell University, Ithaca, NY, USA

Correspondence: David W. Frey (dwf62@cornell.edu)

Supplemental Methods

Soil respiration (Rsoil):

Ambient CO_2 concentration used for flux measurements:

We elected to make all Rsoil measurements at the same ambient CO_2 concentration to standardize measurement conditions across all treatment plots. Measurements were made at an ambient CO_2 concentration of 423 ppm, corresponding to the average measured CO_2 concentration at the soil-atmosphere interface across all plots in July 2020.

Modeling relationship between Rsoil and soil temperature

We used soil temperature and mean Rsoil measurements from all five collars and all 5-6 dates to fit one curve (Eqn. 3, Materials and Methods) for each plot using nonlinear least squares

in R (nls function; R Version 4.4.1). For all sites except the primary forest site at Bald Hill, we used all available soil temperature and Rsoil measurements between October 2020 and October 2021 to model the relationship between soil temperature and Rsoil. In the primary forest stand at the Bald Hill site, Rsoil fluxes from October 2020 were unusually high for the soil temperature, possibly due to a spike in microbial activity stimulated by light rainfall on freshly deposited leaf litter. Inclusion of these measurements led models for this stand to overpredict fluxes at low temperatures, so we elected to remove this month and refit curves for this stand to improve model performance across the range of measured soil temperatures.

Soil moisture can also influence Rsoil (Savage and Davidson, 2001), and it is commonly used to account for residual variation in Rsoil left unexplained by soil temperature (e.g., Carbone et al., 2008; Savage and Davison, 2003; Schedlbauer and Miller, 2022). As such, we used linear and second order polynomial relationships between residuals from exponential Rsoil vs. temperatures models and volumetric soil water content (10 cm depth) to investigate the additional effect of soil water content on Rsoil. Relationships between model residuals and soil moisture were non-significant in all plots for both model forms. Hence, we used temperature alone to predict temporal variation in Rsoil.

Continuous soil temperature measurements and scaling Rsoil measurements to annual fluxes

In July 2020, we installed temperature and moisture sensors at 10 cm depth in the control plots in the three primary forest stands (5TM series at two sites; 5TE series at one site; Decagon Devices; now METER group, Pullman, Washington, USA) interfaced to Campbell Scientific CR1000 data loggers (Campbell Scientific, Logan, Utah, USA). Winter temperature for the Carter Creek site was not available and was estimated from the relationship with soil temperature at the closest site (Bald Hill) during their period of common record. We used a similar approach

to gap-fill brief periods of missing data at each of the other sites (1-day gaps at Bald Hill, < 25-day gaps at Mount Pleasant, Figure S1).

To account for spatial variability in soil temperature within each site we used linear regressions to model the difference between soil temperature at the continuously measured location at each site and the point measurements made at each individual collar. We used these relationships to estimate collar-level temperatures at the hourly timescale between November 1, 2020, and October 31st, 2021 and then calculated plot-level mean hourly temperatures. We used these mean temperatures and fitted parameters from Eqn. 3 (Materials and Methods) to estimate plot-level Rsoil fluxes at an hourly timestep, and summed them for an annual flux.

Soil incubations:

Headspace flushing efficacy, leaks, and leak corrections

Centrifuge tubes used for incubations were fitted with caps equipped with septa to enable gas sampling and rubber gaskets were placed at the interface between caps and tubes to prevent leaks. Three empty centrifuge tubes per stand were included among the soils as negative controls. For blanks and randomly selected, non-blank samples, we collected headspace gas samples prior to incubation (T0 samples) to assess the efficacy of headspace flushing. To collect T0 samples, we first drew a small sample (2-3 mL) from each centrifuge tube and dispensed it into the lab air to flush the needle and stop-cock. We then re-inserted the needle into the septum of the same centrifuge tube, drew ~14-15 mLs of gas from the headspace, and dispensed the sample into a 12 mL evacuated Labco Exetainer® vial (Labco Limited, Lampeter, Wales, UK). After sampling, we immediately drew ~15 mL of CO_2 free air from a centrifuge tube filled with CO_2 free air, and refilled the sample tubes to re-equilibrate vessel pressure. For every ~60 samples from each stand, ten T0 samples were collected.

Analysis of these samples revealed that T0 blanks had a mean CO_2 concentration of 28.7 ppm while T0 values for soils averaged 33.4 ppm, indicating that headspace flushing was sufficient. We calculated leak rates for each incubation batch using T0 and T1 blank CO_2 concentrations. We corrected soil flux values using the mean leak rate for each incubation batch by subtracting batch-level, cumulative mean CO_2 accumulation from leaks from headspace CO_2 concentrations of each sample. Though CO_2 accumulation over time in blanks was small relative to fluxes from soils, they revealed an average leak rate of 8.6 ppm CO_2 hr^{-1} overall (1.4% of the mean forest floor flux, 3.1% of the mean 0-3 cm mineral soil flux, and 6.5% of the mean 3-10 cm mineral soil flux). We further investigated the potential influence of leaks using the δ^{13}C composition of headspace air. δ^{13}C values are calculated as:

$$\left[\frac{\left[\frac{^{13}C}{^{12}C_{sample}}\right]}{\left[\frac{^{13}C}{^{12}C_{standard}}\right]} - 1\right] * 1000 \tag{S1}$$

where $^{13}C/^{12}C_{sample}$ is the measured ratio of ^{13}C to ^{12}C in headspace air, and $^{13}C/^{12}C_{standard}$ is the ratio of ^{13}C to ^{12}C in Vienna Pee Dee Belemnite. The maximum δ^{13}C value in blanks after incubation was -12.82 ‰, and therefore, we excluded 11 samples (nine from field moist incubations and two from incubations with water added to samples from two stands) from the analysis that exceeded this threshold.

Preparing Soils for Second Set of Incubations with Water Added

Precipitation is relatively evenly distributed throughout the year in central NY, but only 65% of average rainfall occurred in June and July 2022 (Game Farm Rd. Weather Station, NRCC, 2024), resulting in dry soils at the last-sampled stands. At the initiation of the field campaign, we elected to incubate soils field moist to avoid variably perturbing soils (i.e., adding water to dryer soils, and/or allowing wetter soils to dry to achieve similar water contents), or

drying all soils and rewetting them prior to incubation, potentially altering microbial C processing dynamics. However, continuous measurements from dataloggers showed that soil moisture declined by 24.7% across sites over the duration of soil collections. Consequently, soils collected from the last two stands were considerably drier than the first four stands, so we conducted an additional set of incubations for these two stands with water added to achieve the mean gravimetric water content measured at the other four stands.

Between the field-moist and moisture-adjusted incubations, we capped soils in centrifuge tubes and kept them refrigerated at 4°C for approximately 1 month. Once removed from refrigeration, we added 1.05 g of DI water to forest floor samples, 1 g of DI water to 0-3 cm mineral soils, and 0.6 g of DI water to 3-10 cm mineral soils. We then preincubated soils for three days rather than one day, as adding water to dry soils can cause large, short-term pulses in microbial activity and CO_2 fluxes (Birch, 1958; Fierer and Schimel, 2003). After preincubation, we followed the same procedure as in the Materials and Methods to measure 24-hour $Rh_{\text{-decomp}}$.

Imputation approach for samples where forest floor depth, soil moisture, or soil carbon data were unavailable

Forest floor depths needed for bulk density estimates were not recorded for 8/95 samples. We estimated depths of these samples using the linear relationship between forest floor depth and fine+coarse forest floor mass across all plots and the mass of the fine+coarse material for the samples where depth data were unavailable. Additionally, soil C and moisture data were unavailable for 15/334 and 8/334 samples, respectively, and we imputed depth-specific plot-level mean values for these samples for statistical analyses.

Soil Nitrogen Availability

Preparing Bags and Resin Beads for Soil Nitrogen Availability Measurements

We constructed bags by placing 5g of Dowex Marathon^{TM} MR-3 mixed bed resin in ~13 x 13 cm squares of nylon swimsuit liner material and sealed them with a zip tie. We activated resin beads by soaking bags in 0.5M HCl for one hour, followed by a rinse with E-pure water. After rinsing, we soaked all bags in 2M NaCl solution for 2-3 hours, after which the solution pH was measured. This process was repeated 5 times, changing out the NaCl solution each time over approximately 12 hours until solution pH stabilized at circumneutral values. Bags were then removed from the NaCl solution, rinsed thoroughly with E-pure water, and refrigerated at 4ºC in plastic bags until field installation (Allison et al., 2008).

Quantifying Resin Available N

At the Cornell Nutrient Analysis Laboratory (CNAL), bags were extracted with 50 mL of 2M KCl and analyzed colorimetrically for NH_4 using the Berthelot reaction. NO_3 + NO_2 concentrations were determined by first reducing extractant nitrate to nitrite with hydrazine, and then subjecting solutions to the Griess reaction. Concentrations of N species were measured using a Bran+Luebbe Automated Ion Analyzer (SPX Flow, Charlotte, NC, USA).

Statistical Methods

Linear mixed effects model forms

There are two general forms of linear mixed effects models used in this study. One set of models include fixed parameters for treatments, stand age (primary vs. secondary), their interaction, and sites, with random effects that allow intercepts to vary by stands (N=6) and plots within stands (N=24). These models can be represented by the equation:

$$Y_{rst} = \beta_0 + \beta_1 Fert_{NO3,rs} + \beta_2 Fert_{AS,rs} + \beta_3 Fert_{S,rs} + \beta_4 Stand_{sec,\,r} + \beta_5 Site_{CC,\,r} + \beta_6 Site_{MP,\,r} + \beta_7 Fert_{NO3} * Stand_{sec,\,rs} + \beta_8 Fert_{AS} * Stand_{sec,\,rs} + \beta_9 Fert_S * Stand_{sec,\,rs} + b_r + \delta_{rs} + \varepsilon_{rst} \quad \text{(S2)}$$

Y_{rst} is the observed value of the tth observation observed in one of *r* stands, in *s* plots, and subjected to one of the *Fert* fertilizer treatments, located within one of two *Stand* age classes that

differ in historical land use and one of three *Sites*. $Fert_{NO3}$ = $NaNO_3$, $Fert_{AS}$ = $(NH_4)_2SO_4$, $Fert_S$ = elemental sulfur, $Stand_{sec}$ = secondary forest, $Site_{CC}$ = Carter Creek, and $Site_{MP}$ = Mt. Pleasant. r = 1-6, s = 1-24, and t varies depending on measurement replication for a given response.

β_0 is the predicted mean in the control treatment in the primary stand at the Bald Hill site;

β_1 is the incremental effect of the $NaNO_3$ treatment;

β_2 is the incremental effect of the $(NH_4)_2SO_4$ treatment;

β_3 is the incremental effect of the elemental sulfur treatment;

β_4 is the incremental effect of the secondary stand;

β_5 is the incremental effect of the Carter Creek site;

β_6 is the incremental effect of the Mount Pleasant site;

β_7 is the further incremental effect of the $NaNO_3$ treatment in the secondary stand;

β_8 is the further incremental effect of the $(NH_4)_2SO_4$ treatment in the secondary stand;

β_9 is the further incremental effect of the elemental sulfur treatment in the secondary stand;

$Fert_{NO3}$ = 1 when the fertilization treatment is $NaNO_3$ and 0 otherwise;

$Fert_{AS}$ = 1 when the fertilization treatment is $(NH_4)_2SO_4$ and 0 otherwise;

$Fert_S$ = 1 when the fertilization treatment is elemental sulfur and 0 otherwise;

$Stand_{sec}$ = 1 when the stand age is secondary and 0 otherwise;

$Site_{CC}$ = 1 when the site is Carter Creek and 0 otherwise;

$Site_{MP}$ = 1 when the site is Mount Pleasant and 0 otherwise;

b_r is the random effect of the rth stand on the response, $b_r \sim N(0, \sigma^2)$, assuming b_r and $b_{r'}$ are independent;

δ_{rs} is the random effect of the sth plot on the response, $\delta_{rs} \sim N(0, \sigma^2)$, assuming δ_{rs} and $\delta_{rs'}$ are independent and;

ε_{rst} is the error associated with the tth observation, $\varepsilon_{rst} \sim N(0, \sigma^2)$, assuming ε_{rst} and $\varepsilon_{rst'}$ are independent. b_r, δ_{rs}, and ε_{rst} are also assumed to be independent.

The second set of models include fixed parameters for the effects of soil pH, resin available N, and sites, and allow intercepts to vary randomly by stand. These models can be represented by the equation:

$$Y_{st} = \beta_0 + \beta_1 soilpH_{st} + \beta_2 \ln(resinN)_{st} + \beta_3 SiteCC_s + \beta_4 SiteMP_s + \delta_s + \varepsilon_{st} \quad (S3)$$

Y_{st} is the observed value of the tth observation (1-24) observed in one of *s* stands (1-6), as a function of soil pH (*soilpH*), the natural log of resin available N (*resinN*), and its location within one of three sites.

β_0 is the mean at the Bald Hill site when soil pH = 0 and resin available N = 1;

β_1 is the effect of a one unit change in soil pH on the response;

β_2 * ln(1.01) is the effect of a one percent increase in resin available N on the response;

β_3 is the incremental effect of the Carter Creek site;

β_4 is the incremental effect of the Mount Pleasant site;

$Site_{CC}$ = 1 when the site is Carter Creek and 0 otherwise;

$Site_{MP}$ = 1 when the site is Mount Pleasant and 0 otherwise;

δ_s is the random effect of the sth stand on the response, $\delta_s \sim N(0, \sigma^2)$, assuming δ_s and $\delta_{s'}$ are independent; and

ε_{st} is the random error associated with the tth observation, $\varepsilon_{st} \sim N(0, \sigma^2)$, assuming ε_{st} and $\varepsilon_{st'}$ are independent. δ_s, and ε_{st} are also assumed to be independent.

Deviations from these general forms are described in the Materials and Methods and below.

Monthly Rsoil model, $Rh_{-decomp}$ C flux models, and models using continuous measures of soil N availability and pH as predictors

We modeled monthly Rsoil using the nlme package (Pinheiro and Bates, 2023) instead of the lme4 package (Bates et al., 2015), as the nlme package supports correlation structures that can be used to account for temporal autocorrelation. The model included all fixed and random parameters described in Eqn. S2, fixed parameters for the interaction between treatments and months to test for differences among treatments over time, fixed parameters for months, and a fixed parameter for soil temperature to account for differences in soil temperature between collars and plots. We also allowed intercepts to vary randomly for collars within plots (N=120) to account for variability associated with multiple measurements at a given collar over time.

We fit three models: 1) a model that did not explicitly account for temporal autocorrelation; 2) a model with constant correlation among residuals of repeated measures (compound symmetry); and 3) a model with unique correlations between all pairs of residuals of repeated measures (general correlation structure). Because the Rsoil data were not collected in evenly spaced time intervals, common covariance structures used in analyses of Rsoil (e.g., AR(1); Bader and Körner, 2010; Lang et al, 2019) were not appropriate. AIC and BIC showed strong support for the model with a general correlation structure, so we used that model for analysis of monthly treatment effects on Rsoil.

For $Rh_{-decomp}$ C flux models where treatments, stand ages, their interaction, and sites were included as predictors (as described in Materials and Methods and shown in Eqn. S2), we also included a fixed parameter to account for variability in soil gravimetric water content.

To understand if resin N availability and soil pH could explain variability in Rsoil and $Rh_{-decomp/SOC}$ across all plots, we constructed linear mixed effects models using natural-log (ln)

transformed plot-level mean resin N availability (2019), plot-level mean soil pH, and site as additive fixed effects and stand as a random effect (Eqn. S3). For the Rsoil model, 2019 0-10 cm mineral soil pH values were used, and for $Rh_{-decomp/SOC}$ models, depth specific (forest floor, 0-3 cm mineral soil, and 3-10 cm mineral soil) soil pH values from 2022 were used. We examined relationships between these variables and annual Rsoil or $Rh_{-decomp/SOC}$ in each soil layer (forest floor, 0-3 cm mineral soils, and 3-10 cm mineral soils). We used the same approach to examine the overall relationship between annual Rsoil and $Rh_{-decomp/m2}$, where Rsoil was the response, $Rh_{-decomp/m2}$ and site were fixed effects, and stand was included as a random effect. All variables from these models were retained in subsequent hierarchical partitioning analyses used to determine marginal R^2 of each predictor.

We assessed model assumptions (linearity, homoskedastic residuals, and residual normality) visually, as recommended in Zuur et al. (2009). Most models met assumptions without need for transformation, but we natural log (resin N, 3-10 cm $Rh_{-decomp/m2}$ from incubations with moisture added) or square root (forest floor $Rh_{-decomp/m2}$ from incubations with moisture added and both forest floor and 3-10 cm $Rh_{-decomp/m2}$ from field moist incubations) transformed response variables if residuals were heteroskedastic across fitted values.

References

Allison, S. D., Czimczik, C. I., & Treseder, K. K. (2008). Microbial activity and soil respiration under nitrogen addition in Alaskan boreal forest. *Global Change Biology*, *14*(5), 1156–1168. https://doi.org/10.1111/j.1365-2486.2008.01549.x

Bader, M. K.-F., & Körner, C. (2010). No overall stimulation of soil respiration under mature deciduous forest trees after 7 years of CO2 enrichment. *Global Change Biology*, *16*(10), 2830–2843. https://doi.org/10.1111/j.1365-2486.2010.02159.x

Bates, D., Maechler, M., Bolker, B., & Walker, S. (2015). Fitting linear mixed-effects models using lme4. *Journal of Statistical Software, 67*(1), 1-48. doi:10.18637/jss.v067.i01.

Birch, H. F. (1958). The effect of soil drying on humus decomposition and nitrogen availability. *Plant and Soil*, *10*(1), 9–31. https://doi.org/10.1007/BF01343734

Carbone, M. S., Winston, G. C., & Trumbore, S. E. (2008). Soil respiration in perennial grass and shrub ecosystems: Linking environmental controls with plant and microbial sources on seasonal and diel timescales. *Journal of Geophysical Research: Biogeosciences*, *113*(G2). https://doi.org/10.1029/2007JG000611

Fierer, N., & Schimel, J. P. (2003). A proposed mechanism for the pulse in carbon dioxide production commonly observed following the rapid rewetting of a dry soil. *Soil Science Society of America Journal*, *67*(3), 798–805. https://doi.org/10.2136/sssaj2003.7980

Lang, A. K., Jevon, F. V., Ayres, M. P., & Hatala Matthes, J. (2020). Higher soil respiration rate beneath arbuscular mycorrhizal trees in a northern hardwood forest is driven by associated soil properties. *Ecosystems*, *23*(6), 1243–1253. https://doi.org/10.1007/s10021-019-00466-7

Northeast Regional Climate Center (NRCC) (2024). Ithaca Climate Page. Cornell University, Ithaca, NY.

Pinheiro, J. & Bates, D. (2023). nlme: Linear and nonlinear mixed effects models. R package version 3.1-164. https://CRAN.R-project.org/package=nlme

Savage, K. E., & Davidson, E. A. (2001). Interannual variation of soil respiration in two New England forests. *Global Biogeochemical Cycles*, *15*(2), 337–350. https://doi.org/10.1029/1999GB001248

Savage, K. E., & Davidson, E. A. (2003). A comparison of manual and automated systems for soil CO2 flux measurements: Trade-offs between spatial and temporal resolution. *Journal of Experimental Botany*, *54*(384), 891–899. https://doi.org/10.1093/jxb/erg121

Schedlbauer, J. L., & Miller, J. (2022). Edge effects increase soil respiration without altering soil carbon stocks in temperate broadleaf forests. *Ecosphere*, *13*(6), e4092. https://doi.org/10.1002/ecs2.4092

Zuur, A. F., Ieno, E. N., Walker, N., Saveliev, A. A., & Smith, G. M. (2009). *Mixed effects models and extensions in ecology with R*. Springer. https://doi.org/10.1007/978-0-387-87458-6
